# Comparative Effectiveness and Safety of Interventions for Atrial Fibrillation in Heart Failure: A Network Meta‐Analysis of Randomized Trials

**DOI:** 10.1155/cdr/6035178

**Published:** 2026-07-07

**Authors:** Changjiang Deng, Yixin Xu, Adilai Adilijiang, Zhilong Wang, Ying Pan, Chao Fan, Baichuan Liu, Haoxu Lv, Junrui Hou, Tingting Wu

**Affiliations:** ^1^ Department of Cardiology, First Affiliated Hospital of Xinjiang Medical University, Urumqi, Xinjiang, China, xjmu.edu.cn; ^2^ Department of Cardiology, Southwest Hospital of AMU, Chongqing, Chongqing, China; ^3^ Department of Pathophysiology, Xinjiang Medical University College of Basic Medicine, Urumqi, Xinjiang, China

**Keywords:** atrial fibrillation, atrioventricular node ablation, cardiac resynchronization therapy, catheter ablation, heart failure, network meta-analysis

## Abstract

**Background:**

Patients with HF and AF face high risks of death and HF decompensation, yet the comparative effectiveness of commonly used AF management strategies remains uncertain. This study is aimed at comparing the efficacy and safety of different interventional and medical approaches using NMA of RCTs.

**Methods:**

PubMed, Embase, CENTRAL, Web of Science, and Google Scholar were searched from inception to November 30, 2025, without language restrictions. Parallel‐group RCTs enrolling adults with clinically diagnosed HF and documented AF were eligible if they compared at least two AF management strategies, including CA, AVNA with CRT or RV pacing, MRC, drug‐based rhythm control (rhythm drug), or CON. Primary outcomes were all‐cause mortality and HF hospitalization; secondary outcomes were worsening HF, CV mortality, QoL, and LVEF. Random‐effects NMA was performed in Stata 17.0, reporting ORs and SMDs with 95% CIs; treatments were ranked using SUCRA.

**Results:**

Twenty‐three RCTs (5159 patients) were included. For all‐cause mortality (21 trials; 5095 patients), AVNA_CRT (OR, 0.30; 95% CI, 0.12–0.78) and CA (OR, 0.53; 95% CI, 0.35–0.82) were associated with lower mortality than CON; AVNA_CRT also outperformed MRC (OR, 0.35; 95% CI, 0.15–0.79) and AVNA_RV (OR, 0.49; 95% CI, 0.25–0.96). For HF hospitalization (9 trials; 3491 patients), AVNA_CRT reduced risk compared with AVNA_RV (OR, 0.20; 95% CI, 0.04–0.94). For worsening HF (7 trials; 2542 patients), AVNA_CRT was superior to AVNA_RV (OR, 0.41; 95% CI, 0.20–0.84). CA improved QoL versus AVNA_CRT (SMD, −0.94; 95% CI, −1.83 to −0.05) and MRC (SMD, −0.73; 95% CI, −1.22 to −0.24), and improved LVEF versus MRC (SMD, 1.49; 95% CI, 0.57–2.41). No between‐strategy differences were clearly demonstrated for CV mortality.

**Conclusion:**

In RCT evidence for HF with AF, AVNA_CRT and CA were associated with more favorable prognostic outcomes, and CA provided more consistent improvements in QoL and LVEF. When AVNA is selected, CRT should be preferred over RV pacing. Strategy choice should be individualized to clinical goals, patient phenotype, and procedural feasibility.

## 1. Introduction

Heart failure (HF) and atrial fibrillation (AF) frequently coexist and together define a high‐risk clinical phenotype marked by substantial symptom burden, impaired functional capacity, recurrent hospitalizations, and excess mortality. AF is documented in approximately one third of patients with chronic HF and in about 44% of those with acute HF, with prevalence rising as HF severity increases [1]. In contemporary population‐based analyses, concomitant HF in patients with AF has been associated with nearly a doubling of all‐cause mortality [2]. The association is bidirectional. AF can worsen HF through rapid and irregular ventricular activation, loss of atrial contribution to ventricular filling, and reduced cardiac efficiency, whereas HF promotes AF through atrial stretch, fibrosis, inflammation, and electrophysiologic remodeling [3]. As a result, HF complicated by AF represents a common and consequential therapeutic challenge in which the optimal strategy to improve prognosis and patient‐centered outcomes remains uncertain.

Contemporary management of AF in patients with HF includes pharmacologic rate control, drug‐based rhythm control with antiarrhythmic drugs (AADs), catheter ablation (CA) aimed at durable rhythm control, and atrioventricular node ablation (AVNA) with permanent pacing, often with cardiac resynchronization therapy (CRT) [4]. Each approach has plausible mechanistic advantages and distinct tradeoffs. Rate control may reduce symptoms and avoid procedure‐related risks but can fail to prevent tachycardia‐mediated cardiomyopathy and may not restore atrial mechanical function [5]. Drug‐based rhythm control can maintain sinus rhythm in selected patients but is limited by incomplete efficacy and adverse effects, particularly in HF populations [6]. CA can reduce AF burden and potentially improve ventricular function and quality of life (QoL), but procedural complexity, recurrence, and heterogeneous ablation strategies complicate interpretation across trials [7]. AVNA with pacing secures ventricular rate regularity and, when paired with CRT, may mitigate desynchrony, yet outcomes may differ meaningfully between biventricular pacing and right ventricular (RV) pacing [8].

Randomized controlled trials (RCTs) have evaluated these strategies in HF with AF, but the evidence base remains difficult to translate into clear clinical choices. Trials differ in HF phenotype and severity, AF type and chronicity, background guideline directed therapy, endpoint definitions, and duration of follow up [9]. Many studies compare only two strategies, commonly CA versus medical therapy or CRT‐based pacing versus RV pacing after AVNA [10]. Conventional pairwise meta‐analyses have therefore been constrained to limited comparisons and have rarely provided a unified assessment of competing strategies across hard clinical outcomes and patient‐reported measures [11, 12]. Moreover, clinicians often need to balance survival and hospitalization risk against improvements in left ventricular function and QoL, yet prior syntheses have not consistently integrated these domains within a single comparative framework.

Network meta‐analysis (NMA) can combine direct and indirect evidence to estimate relative effects among multiple interventions and to support evidence‐based ranking while preserving the randomized structure of included trials. Accordingly, the present study performed a systematic review and NMA of RCTs in adults with HF and AF to compare the efficacy and safety of commonly used management strategies across all‐cause mortality, HF hospitalization, worsening HF, cardiovascular (CV) mortality, QoL, and left ventricular ejection fraction (LVEF), with the goal of informing clinical decision‐making in this high‐risk population.

## 2. Methods

### 2.1. Protocol and Reporting

This systematic review and NMA was conducted in accordance with the Preferred Reporting Items for Systematic Reviews and Meta Analyses (PRISMA) statement and the PRISMA extension for NMA. The protocol was registered in the International Prospective Register of Systematic Reviews (PROSPERO CRD420261284417) (Supporting Material 1) [13].

### 2.2. Data Sources and Search Strategy

PubMed, Embase, the Cochrane Central Register of Controlled Trials (CENTRAL), Web of Science, and Google Scholar were searched from inception to November 30, 2025, without language restrictions. The search combined controlled vocabulary and free text terms for HF, AF, and AF management strategies, including CA (pulmonary vein isolation [PVI] with or without additional ablation), AVNA with pacing or CRT, and pharmacologic rate or rhythm control. Full search strategies and search dates are provided in Supporting Material 2. Reference lists of relevant RCTs and reviews were screened to identify additional eligible studies.

### 2.3. Study Selection

Records were imported into EndNote X9 and deduplicated. Two reviewers independently screened titles and abstracts and then assessed full texts. Disagreements were resolved by discussion, with adjudication by a third reviewer when necessary.

### 2.4. Eligibility Criteria

Eligible studies were parallel group RCTs enrolling adults (≥ 18 years) with clinically diagnosed HF and documented AF (paroxysmal, persistent, or long‐standing persistent) confirmed by electrocardiography (ECG), ambulatory monitoring, or implanted device interrogation, and comparing at least two prespecified AF management strategies, including (a) CA; (b) AVNA plus CRT or RV pacing; (c) medical rate control (MRC); (d) drug‐based rhythm control with AADs, with cardioversion permitted when prespecified; and (e) conventional or usual care AF management. Studies were required to report at least one prespecified efficacy or safety outcome.

Studies were excluded if they were nonrandomized, single‐arm, lacked a concurrent comparator, or were protocols, conference abstracts without analyzable data, reviews, meta‐analyses, editorials, case reports, animal studies, or in vitro studies. Additional exclusions were trials without an HF population or without explicit reporting of HF status, trials restricted to perioperative or postoperative new‐onset transient AF, and trials evaluating only antithrombotic strategies without comparing AF management approaches.

### 2.5. Data Extraction and Processing

Two reviewers independently extracted study characteristics, baseline participant features (including New York Heart Association [NYHA] class, LVEF, AF type, and duration), intervention details, follow‐up duration, and outcomes; discrepancies were resolved by consensus. For continuous outcomes, means and standard deviations (SDs) were extracted; when standard errors (SEs) were reported, SDs were calculated as SD = SE × √n. When required, SDs were estimated from confidence intervals (CIs), *p* values, *t* statistics, ranges, or interquartile ranges (IQRs) using methods recommended in the Cochrane Handbook. When essential data were unavailable, corresponding authors were contacted at least four times over 6 weeks. No formal imputation was performed for missing baseline descriptive variables; such data were recorded as not reported (NR). Intention to treat (ITT) estimates were preferentially extracted.

### 2.6. Intervention Nodes

Interventions were grouped into prespecified nodes to support transitivity: CA, atrioventricular node ablation plus CRT (AVNA_CRT), atrioventricular node ablation plus RV pacing (AVNA_RV), MRC, drug based rhythm control (RhythmDrug), and conventional care (CON). Combined regimens were classified according to the randomized strategy specified in the trial protocol.

### 2.7. Outcomes

Primary outcomes were all‐cause mortality and HF hospitalization. Secondary outcomes included worsening HF, CV mortality, QoL assessed with validated instruments, including the Minnesota Living with Heart Failure Questionnaire (MLHFQ), Kansas City Cardiomyopathy Questionnaire (KCCQ), and Atrial Fibrillation Effect on Quality of Life (AFEQT), and LVEF measured by standard imaging modalities. Because these instruments use different scoring systems, QoL outcomes were synthesized as standardized mean differences (SMDs) after harmonizing score direction across studies. When outcomes were reported at multiple time points, the longest randomized follow‐up was used.

### 2.8. Risk of Bias Assessment

Two reviewers assessed risk of bias using the revised Cochrane Risk of Bias tool (RoB 2), addressing the randomization process, deviations from intended interventions, missing outcome data, outcome measurement, and selective reporting [14]; disagreements were resolved by a third reviewer.

### 2.9. Statistical Analysis

All analyses were performed in Stata (Version 17.0; StataCorp) using a frequentist framework. A random effects NMA was used to estimate comparative effects while accounting for within‐study and between‐study variability, because clinical and methodological heterogeneity was anticipated across trials, including differences in HF phenotype and severity, AF type, intervention implementation, and follow‐up duration. Binary outcomes were summarized as odds ratios (ORs) with 95% CIs, and continuous outcomes were summarized as SMDs with 95% CIs. Network plots were generated to describe the evidence structure.

Heterogeneity was assessed using the *I* ^2^ statistic in pairwise meta‐analyses and by the estimated between study variance in network models; *I* ^2^ values of 25%, 50%, and 75% were interpreted as low, moderate, and high heterogeneity. Transitivity was examined by comparing potential effect modifiers across treatment comparisons, including age, baseline LVEF, NYHA class, AF phenotype, and follow up duration. Consistency was assessed at both the global and local levels. Global inconsistency was evaluated using the design by treatment interaction model, and local inconsistency was examined using node‐splitting analyses when the network geometry allowed direct and indirect evidence to be separated for the same comparison. Detailed results of inconsistency testing are provided in Supporting Materials 6 and 7.

Treatment ranking was summarized using the surface under the cumulative ranking curve (SUCRA). Small study effects were explored with comparison adjusted funnel plots and Egger tests when sufficient studies were available, and prediction intervals were used to contextualize heterogeneity [15]. All tests were two sided, and *p* < 0.05 was considered statistically significant. Sensitivity analyses excluding trials judged as having some concerns in the overall RoB 2 assessment were performed for the primary outcomes when the reduced network remained estimable. Formal subgroup analyses and metaregression were not performed because the number of studies within most network contrasts was limited and the network geometry was sparse, which was considered unlikely to support stable or reliable estimates.

## 3. Results

### 3.1. Characteristics of the Included Studies

**Figure 1:**
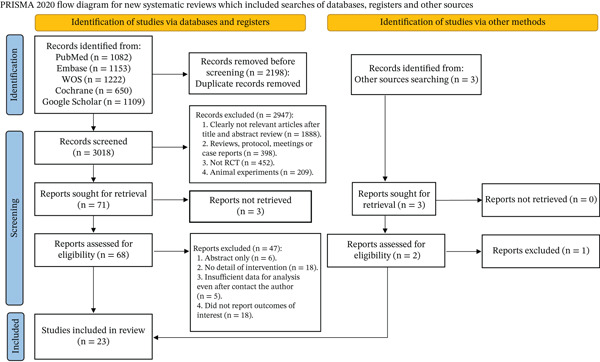
PRISMA flow diagram of study selection.

The initial electronic search identified 5216 records. After removal of 2198 duplicates, 3018 records underwent title and abstract screening. Of these, 2947 were excluded, and 71 full‐text articles were assessed for eligibility. Ultimately, 23 RCTs involving 5159 patients with HF and AF were included in the systematic review and NMA (Figure 1) [16–38].

The included trials were published between 1998 and 2025, with a median publication year of 2017. Six trials were multinational collaborations, four were conducted in the United Kingdom, and two were conducted in Australia. Three trials were single‐blinded RCTs and 20 were open‐label RCTs. Sample sizes ranged from 41 to 1376 participants, with a median of 133 participants per trial. Across studies, mean age ranged from 55 to 72 years, with a median of 65 years.

Regarding intervention strategies, five trials evaluated AVNA_CRT and three evaluated AVNA_RV. Fourteen trials investigated CA. Five trials assessed RDC, 13 assessed MRC, and six included CON as a comparator. Detailed characteristics of the included studies are provided in Supporting Material 3.

### 3.2. Results of NMA

#### 3.2.1. Primary Outcomes

##### 3.2.1.1. All‐Cause Mortality

**Figure 2:**
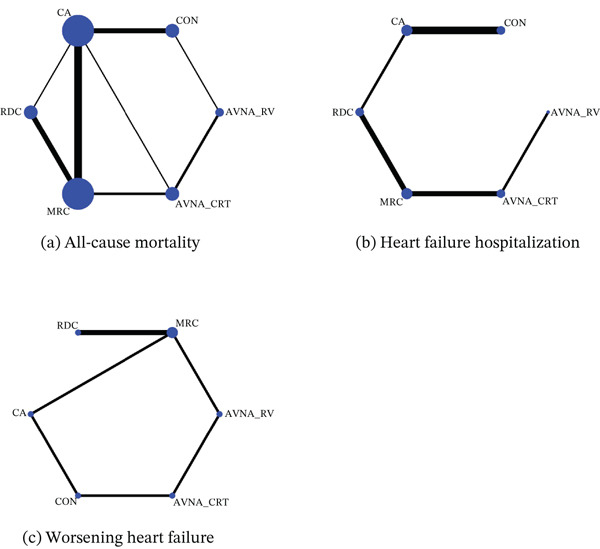
Network geometry for primary outcomes: (a) all‐cause mortality, (b) heart failure hospitalization, and (c) worsening heart failure. Node size reflects sample size; edge thickness reflects the number of direct comparisons.

**Figure 3:**
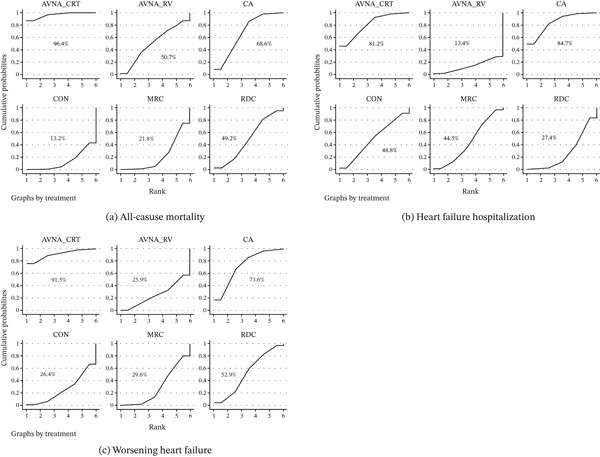
SUCRA rankings for primary outcomes: (a) all‐cause mortality, (b) heart failure hospitalization, and (c) worsening heart failure.

Twenty‐one trials (5095 patients with HF and AF) reported all‐cause mortality. The evidence network for all‐cause mortality, including direct comparisons and sample size distribution, is shown in Figure 2a. Based on treatment ranking using the SUCRA (Figure 3a), the three highest‐ranked strategies for reducing all‐cause mortality were AVNA_CRT (96.4%), CA (68.6%), and AVNA_RV (50.7%), whereas CON had the lowest ranking (13.2%).

Consistent with these rankings, AVNA_CRT (OR, 0.30; 95% CI, 0.12–0.78) and CA (OR, 0.53; 95% CI, 0.35–0.82) were each associated with significantly lower all‐cause mortality than CON (Table 1). AVNA_CRT was also superior to MRC (OR, 0.35; 95% CI, 0.15–0.79) and AVNA_RV (OR, 0.49; 95% CI, 0.25–0.96) in reducing all‐cause mortality (Table 1).

**Table 1:** League table of primary outcomes in participants.

All‐cause mortality					
AVNA_CRT					
0.56 (0.23, 1.39)	CA				
**0.49 (0.25, 0.96)**	0.86 (0.31, 2.40)	AVNA_RV			
0.45 (0.18, 1.16)	0.81 (0.44, 1.47)	0.93 (0.32, 2.73)	RDC		
**0.35 (0.15, 0.79)**	0.62 (0.37, 1.03)	0.72 (0.27, 1.91)	0.77 (0.47, 1.24)	MRC	
**0.30 (0.12, 0.78)**	**0.53 (0.35, 0.82)**	0.62 (0.22, 1.76)	0.66 (0.32, 1.37)	0.86 (0.45, 1.66)	CON

*Note:* Bold values indicate statistically significant differences (95% CI excluding 1.0 for odds ratios; 95% CI excluding 0 for standardized mean differences).

##### 3.2.1.2. HF Hospitalization

Nine trials (3491 patients with HF and AF) reported HF hospitalization. The corresponding network structure, including direct comparisons and sample size distribution, is shown in Figure 2b. SUCRA rankings for reducing HF hospitalization (Figure 3b) indicated that CA (84.7%) and AVNA_CRT (81.2%) were the top two strategies, followed by CON (48.8%), whereas AVNA_RV ranked lowest (13.4%).

In comparative estimates (Table 2), AVNA_CRT was associated with a significantly lower risk of HF hospitalization than AVNA_RV (OR, 0.20; 95% CI, 0.04–0.94).

**Table 2:** Heart failure hospitalization.

CA					
0.95 (0.19, 4.65)	AVNA_CRT				
0.57 (0.32, 1.03)	0.60 (0.11, 3.26)	CON			
0.49 (0.13, 1.85)	0.52 (0.21, 1.26)	0.86 (0.20, 3.61)	MRC		
0.38 (0.13, 1.12)	0.40 (0.12, 1.29)	0.67 (0.19, 2.27)	0.78 (0.36, 1.68)	RDC	
0.19 (0.02, 1.76)	**0.20 (0.04, 0.94)**	0.34 (0.03, 3.30)	0.40 (0.07, 2.32)	0.51 (0.07, 3.48)	AVNA_RV

*Note:* Bold values indicate statistically significant differences (95% CI excluding 1.0 for odds ratios; 95% CI excluding 0 for standardized mean differences).

##### 3.2.1.3. Worsening HF

Seven trials (2542 patients with HF and AF) reported worsening HF. The network plot with direct comparisons and sample size distribution is presented in Figure 2c. SUCRA rankings (Figure 3c) suggested that AVNA_CRT (91.5%) and CA (73.6%) were most likely to reduce worsening HF, followed by RDC (52.9%), whereas AVNA_RV ranked lowest (25.9%).

As shown in Table 3, AVNA_CRT significantly reduced worsening HF compared with AVNA_RV (OR, 0.41; 95% CI, 0.20–0.84).

**Table 3:** Worsening heart failure.

AVNA_CRT					
0.68 (0.26, 1.79)	CA				
0.55 (0.21, 1.44)	0.80 (0.48, 1.35)	RDC			
0.47 (0.18, 1.22)	0.70 (0.44, 1.11)	0.87 (0.69, 1.10)	MRC		
0.42 (0.16, 1.10)	0.62 (0.31, 1.25)	0.77 (0.34, 1.77)	0.89 (0.40, 1.98)	CON	
**0.41 (0.20, 0.84)**	0.60 (0.23, 1.57)	0.75 (0.28, 2.04)	0.86 (0.33, 2.29)	0.97 (0.42, 2.27)	AVNA_RV

*Note:* Bold values indicate statistically significant differences (95% CI excluding 1.0 for odds ratios; 95% CI excluding 0 for standardized mean differences).

#### 3.2.2. Secondary Outcomes

##### 3.2.2.1. QoL

**Figure 4:**
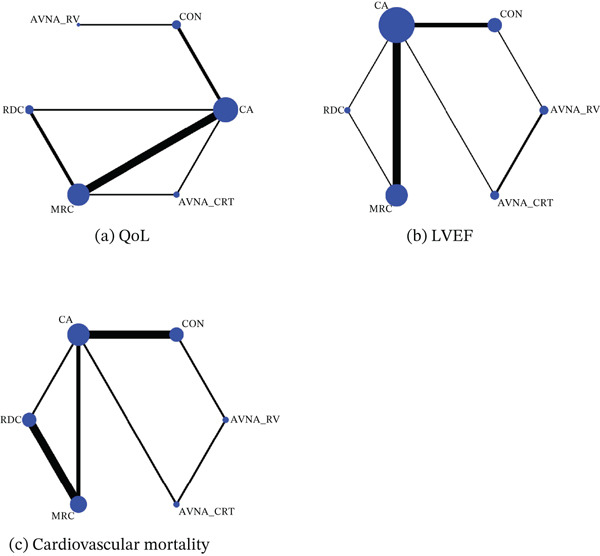
Network geometry for secondary outcomes: (a) quality of life, (b) left ventricular ejection fraction, and (c) cardiovascular mortality. Node size reflects sample size; edge thickness reflects the number of direct comparisons.

**Figure 5:**
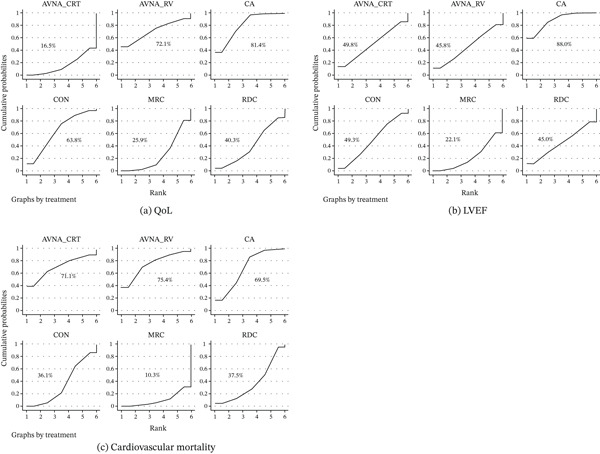
SUCRA rankings for secondary outcomes: (a) quality of life, (b) left ventricular ejection fraction, and (b) cardiovascular mortality.

Thirteen trials (2312 patients with HF and AF) reported QoL outcomes, measured mainly with MLHFQ, KCCQ, and AFEQT. The evidence network for QoL is shown in Figure 4a. According to SUCRA rankings (Figure 5a), CA (81.4%) was most likely to improve QoL, followed by AVNA_RV (72.1%) and CON (63.8%); the lowest‐ranked strategy was AVNA_CRT (16.5%).

In pairwise comparisons derived from the NMA (Table 4), CA was associated with significantly better QoL than AVNA_CRT (SMD, −0.94; 95% CI, −1.83 to −0.05) and MRC (SMD, −0.73; 95% CI, −1.22 to −0.24). Because QoL was pooled across different instruments, these SMDs should be interpreted as standardized effects rather than directly mapped to a single instrument‐specific threshold.

**Table 4:** League table of secondary outcomes in participants.

QoL					
CA					
0.02 (−1.44, 1.48)	AVNA_RV				
−0.20 (−1.00, 0.61)	−0.22 (−1.43, 1.00)	CON			
−0.56 (−1.30, 0.19)	−0.58 (−2.22, 1.06)	−0.36 (−1.46, 0.74)	RDC		
**−0.73 (−1.22, −0.24)**	−0.75 (−2.29, 0.78)	−0.54 (−1.48, 0.41)	−0.18 (−0.87, 0.52)	MRC	
**−0.94 (−1.83, −0.05)**	−0.96 (−2.66, 0.75)	−0.74 (−1.94, 0.46)	−0.38 (−1.47, 0.71)	−0.20 (−1.09, 0.68)	AVNA_CRT

*Note:* Bold values indicate statistically significant differences (95% CI excluding 1.0 for odds ratios; 95% CI excluding 0 for standardized mean differences).

##### 3.2.2.2. LVEF

Seventeen trials (2448 patients with HF and AF) reported LVEF. The network plot for LVEF is shown in Figure 4b. SUCRA rankings for improving LVEF (Figure 5b) indicated that CA ranked highest (88.0%), followed by AVNA_CRT (49.8%) and CON (49.3%), whereas MRC ranked lowest (22.1%).

Consistent with these rankings, CA significantly improved LVEF compared with MRC (SMD, 1.49; 95% CI, 0.57–2.41) (Table 5).

**Table 5:** LVEF.

CA					
0.85 (−1.13, 2.83)	AVNA_CRT				
0.87 (−0.31, 2.06)	0.02 (−2.03, 2.07)	CON			
0.95 (−1.11, 3.00)	0.09 (−1.49, 1.68)	0.08 (−1.91,2.06)	AVNA_RV		
0.96 (−0.85,2.76)	0.10 (−2.58, 2.78)	0.08 (−2.08, 2.25)	0.01 (−2.73, 2.75)	RDC	
**1.49 (0.57, 2.41)**	0.64 (−1.54, 2.82)	0.62 (−0.88, 2.11)	0.54 (−1.71, 2.79)	0.53 (−1.28, 2.34)	MRC

*Note:* Bold values indicate statistically significant differences (95% CI excluding 1.0 for odds ratios; 95% CI excluding 0 for standardized mean differences).

##### 3.2.2.3. CV Mortality

Fourteen trials (3952 patients with HF and AF) reported CV mortality. The evidence network is shown in Figure 4c. Based on SUCRA rankings (Figure 5c), the three highest‐ranked strategies for reducing CV mortality were AVNA_RV (75.4%), AVNA_CRT (71.1%), and CA (69.5%), whereas MRC ranked lowest (10.3%). However, none of the between‐group comparisons demonstrated statistically significant differences in CV mortality (Table 6).

**Table 6:** Cardiovascular mortality.

AVNA_RV					
0.96 (0.07, 13.09)	AVNA_CRT				
0.60 (0.05, 6.56)	0.62 (0.03, 11.37)	CA			
0.25 (0.01, 4.64)	0.26 (0.01, 7.42)	0.42 (0.08, 2.22)	RDC		
0.30 (0.03, 3.01)	0.31 (0.02, 5.62)	0.50 (0.23, 1.09)	1.19 (0.19, 7.58)	CON	
0.13 (0.01, 2.48)	0.13 (0.00, 3.96)	0.22 (0.04, 1.21)	0.52 (0.22, 1.27)	0.44 (0.07, 2.91)	MRC

#### 3.2.3. Assessment of Inconsistency

Global inconsistency was assessed using the design by treatment interaction model. No significant global inconsistency was detected for all‐cause mortality (*p* = 0.389), HF hospitalization (*p* = 0.519), worsening HF (*p* = 0.725), LVEF (*p* = 0.928), or CV mortality (*p* = 0.735). In contrast, the QoL network showed evidence of global inconsistency (*p* = 0.018). Local inconsistency was further explored using node‐splitting analyses. No significant disagreement between direct and indirect evidence was observed for all‐cause mortality, HF hospitalization, worsening HF, LVEF, or CV mortality. For QoL, two comparisons showed significant local inconsistency (both *p* = 0.004), whereas the remaining comparisons were not statistically significant. Detailed results are presented in Supporting Materials 6 and 7.

### 3.3. Risk of Bias and Publication Bias

Across the 23 RCTs, 14 trials were judged to be at low overall risk of bias and nine were judged to have some concerns; no study was rated as high risk. For the randomization process, 21 trials were rated as low risk and two as some concerns. For deviations from intended interventions, 19 trials were rated as low risk and four as some concerns. For missing outcome data, 16 trials were rated as low risk and seven as some concerns. For outcome measurement and selection of the reported result, all 23 trials were judged as low risk (Supporting Material 4). Overall, the main sources of potential bias were deviations from intended interventions and missing outcome data. Sensitivity analyses excluding studies with some concerns showed that the direction of effect for all‐cause mortality was largely preserved. In particular, CA versus CON remained significant (OR, 0.58; 95% CI, 0.37–0.91), as did AVNA_CRT versus MRC (OR, 0.40; 95% CI, 0.19–0.87), whereas AVNA_CRT versus CON was attenuated and no longer statistically significant (OR, 0.43; 95% CI, 0.18–1.03). Comparisons involving AVNA_RV were no longer estimable after exclusion of studies with some concerns. For HF hospitalization, the reduced network showed no statistically significant differences between strategies (Supporting Material 8).

Potential publication bias was evaluated using funnel plots and Egger′s regression tests (Supporting Material 5). Visual inspection suggested varying degrees of asymmetry across the outcome networks, indicating possible small‐study effects. Egger′s regression tests showed no statistically significant asymmetry for all‐cause mortality (coefficient, 0.5186853; SE, 0.5031169; *p* = 0.316), HF hospitalization (coefficient, 0.9111647; SE, 1.029242; *p* = 0.405), worsening HF (coefficient, 0.9114135; SE, 0.9198344; *p* = 0.367), QoL (coefficient, −1.812363; SE, 2.212187; *p* = 0.430), LVEF (coefficient, −3.922308; SE, 5.096026; *p* = 0.453), or CV mortality (coefficient, 0.1541886; SE, 0.5946334; *p* = 0.800). However, given the limited number of studies for several outcomes, these findings do not exclude the possibility of small‐study effects.

## 4. Discussion

In this systematic review and NMA of RCTs in patients with HF and AF, three main findings have direct implications for clinical decision‐making. First, AVNA_CRT and CA were generally associated with more favorable prognostic outcomes, with the most consistent advantage observed for all‐cause mortality. Second, within AVNA‐based strategies, AVNA_CRT outperformed AVNA_RV for HF events, including hospitalization and worsening HF, indicating that the clinical value of AVNA is tightly coupled to the pacing modality, and that ventricular resynchronization may be the key determinant of benefit. Third, CA showed more prominent improvements in QoL and LVEF, whereas no clear between‐strategy differences were observed for CV mortality in the available evidence. By integrating direct and indirect comparisons within a single randomized evidence framework, this NMA enables coherent cross‐strategy inference and clarifies the relative tradeoffs between hard endpoints and patient‐centered outcomes, thereby strengthening the evidence base for individualized management in this high‐risk population. These findings provide the foundation for examining why AVNA_CRT and CA may translate into improved prognosis. At the same time, several primary outcome estimates, particularly for HF hospitalization and some all‐cause mortality comparisons, were accompanied by wide CIs. This suggests limited precision rather than absence of effect, and these nonsignificant findings should therefore be interpreted cautiously in clinical decision making.

The prognostic signal favoring AVNA_CRT and CA align with, and may help reconcile, the mixed RCT literature in HF with AF. For CA, landmark trials in symptomatic HF populations, including CASTLE‐AF and AATAC, suggested that an ablation‐based strategy can reduce major clinical events, whereas other randomized evidence, including subgroup analyses from broader trials such as CABANA, has shown more variable effects on hard endpoints despite improvements in rhythm control and patient‐reported outcomes [28, 39]. Such inconsistency is plausibly driven by differences in case mix and trial design, including HF phenotype and severity, AF chronicity, baseline LVEF, procedural technique and lesion sets, intensity of background GDMT, and the choice and adjudication of clinical endpoints [40]. These factors may have influenced not only the magnitude of treatment effect but also the likelihood that benefit would be captured as symptomatic improvement, reverse remodeling, or hard clinical events. In particular, shorter follow‐up may be more likely to detect changes in QoL or LVEF, whereas mortality and HF hospitalization may require longer observation to reflect durable treatment effects. In this context, the present NMA supports the interpretation that CA may translate mechanistically plausible benefits into prognostic gains in appropriately selected patients by reducing AF burden and ventricular irregularity, improving ventricular filling and cardiac output, lowering myocardial oxygen demand and neurohormonal activation, and promoting reverse remodeling over time [41]. Randomized evidence supporting an AVNA plus CRT strategy, exemplified by APAF‐CRT and related trials, indicates that definitive rate control coupled with consistent biventricular pacing can improve clinical outcomes in patients with permanent AF and HF, particularly when pharmacologic rate control is ineffective or poorly tolerated [18]. Biologically, AVNA provides stable ventricular rate control and facilitates near‐complete biventricular capture, thereby reducing beat‐to‐beat variability, improving ventricular synchrony, and potentially mitigating progressive remodeling and HF decompensation [8]. These hemodynamic effects are sustained by design and may be especially relevant in long‐standing AF, in which durable rhythm restoration is less likely and a reliable strategy to stabilize ventricular performance is required to influence mortality [42]. The current findings suggest that prognostic benefit in HF with AF may be concentrated in strategies that either modify the arrhythmic substrate (CA) or deliver durable rate regularization with ventricular resynchronization (AVNA_CRT), setting the stage for a closer examination of how pacing modality modifies outcomes after AVNA.

The finding that AVNA_CRT outperformed AVNA_RV for HF events underscores that AVNA is not a uniform intervention and that its clinical effect is strongly conditioned by the pacing strategy that follows. AVNA achieves definitive rate control by creating complete atrioventricular block, but it simultaneously renders patients pacing dependent, often with a near 100% ventricular pacing burden. Under these circumstances, chronic RV apical pacing can impose an iatrogenic pattern of electrical activation that mimics left bundle‐branch block, leading to interventricular and intraventricular desynchrony, adverse remodeling, and declines in systolic function [43]. This biologic framework is supported by prior pacing literature linking a high RV pacing burden to pacing‐induced cardiomyopathy and increased HF risk, particularly in patients with preexisting ventricular dysfunction [44]. In HF with AF, these deleterious effects may plausibly offset the hemodynamic gains from rate regularization achieved by AVNA, which provides a mechanistic explanation for less favorable HF‐related outcomes with AVNA_RV [45]. By contrast, CRT is designed to prevent or correct desynchrony by coordinating biventricular activation, an effect that becomes clinically salient when pacing is frequent or obligatory. Randomized trials and meta‐analytic evidence in patients with HF, as well as guidance for patients who require a high percentage of ventricular pacing, support CRT as the preferred pacing modality to preserve or improve ventricular function and reduce HF events [46]. In AF populations undergoing AVNA, CRT can also facilitate consistently high biventricular capture, thereby converting the stable rhythm environment created by AVNA into sustained mechanical synchrony and more favorable loading conditions [47]. The present findings reinforce a practical principle for strategy selection: AVNA should not be viewed as interchangeable across pacing modalities, and the combination of AVNA with CRT is more likely than AVNA with RV pacing to translate rate regularization into reduced HF decompensation. This distinction also provides a useful bridge to the next question raised by the current analysis, namely why CA appeared to confer more prominent improvements in QoL and LVEF.

CA showed more prominent benefits in QoL and LVEF than the other strategies, a pattern that is consistent with prior RCTs and systematic reviews in HF with AF, in which functional and symptom‐related gains have tended to be more reproducible than effects on mortality [39]. Mechanistically, QoL and LVEF are relatively sensitive intermediate outcomes that can improve with reductions in AF burden and ventricular irregularity, better diastolic filling, and favorable loading conditions that promote reverse remodeling [48]. Because QoL was synthesized across MLHFQ, KCCQ, and AFEQT using SMDs, the pooled estimates cannot be directly translated into a single minimal important difference. Nevertheless, the magnitude of benefit observed with CA is consistent with a clinically meaningful improvement, although some caution is warranted given instrument heterogeneity. This caution is reinforced by the fact that inconsistency was confined to the QoL network, in which both global and local inconsistency were detected; accordingly, the apparent advantage of CA for QoL should be interpreted more cautiously than the mortality findings. In contrast, MRC and rhythm drug often yield incomplete rhythm stabilization, are limited by adverse effects and discontinuation, and may be less likely to produce sustained reductions in AF burden or consistent reverse remodeling, which could explain their comparatively smaller effects on these outcomes [41]. The absence of clear between‐strategy differences in CV mortality should be interpreted cautiously. CV death is a less frequent and more heterogeneously defined endpoint across trials, and the available evidence is influenced by differences in follow‐up duration, event ascertainment and adjudication, competing risks from no CV death, and clinical heterogeneity in HF severity and AF chronicity. Accordingly, a nonsignificant finding does not exclude clinically meaningful differences but indicates limited discriminative power of the current evidence base for this terminal outcome. Overall, these results suggest that CA may offer more immediate, patient‐centered and functional benefits, whereas prognostic separation across strategies is more likely to emerge through durable hemodynamic stabilization and remodeling, which informs how these tradeoffs should be weighed in practice.

These results suggest an individualized decision framework for HF with AF that balances prognostic goals (all‐cause mortality and HF events) against patient‐centered benefits (QoL and LVEF) and aligns treatment with phenotype, symptom burden, and feasibility of durable implementation. AVNA_CRT may be favored when the primary objective is to reduce HF decompensation and improve prognosis in patients with refractory or poorly tolerated pharmacologic rate control, long‐standing or permanent AF, or an anticipated high pacing burden. CA may be prioritized when symptom relief and functional improvement are dominant goals and when procedural candidacy and patient preference support an interventional rhythm‐control strategy, given its more consistent association with improvements in QoL and LVEF. When an AVNA pathway is selected, pacing modality should be treated as a key determinant of benefit, with CRT preferred over RV pacing rather than viewed as interchangeable.

This study has several strengths. By restricting inclusion to RCTs and using an NMA framework, it enabled simultaneous comparison of multiple commonly used strategies and provided a coherent synthesis of direct and indirect evidence. The evaluation encompassed both major clinical endpoints (mortality and HF events) and patient‐centered or functional outcomes (QoL and LVEF), which improves interpretability for real‐world decision making. In addition, the prespecified intervention nodes and outcome set reflected pragmatic clinical pathways in HF with AF, supporting translation to practice and future guideline discussions.

Several limitations should be considered. First, clinical and methodological heterogeneity across trials was substantial, including variation in HF phenotype and severity, AF type and duration, ablation technique and lesion sets, background GDMT, follow‐up length, and endpoint definitions and ascertainment; these differences can challenge transitivity and contribute to residual inconsistency despite a random‐effects approach. They may also have influenced how treatment benefits were expressed, with shorter follow‐up more likely to capture changes in QoL or LVEF than in mortality or HF hospitalization. Although no study was judged to be at high risk of bias, nine trials were rated as having some concerns, mainly related to deviations from intended interventions and missing outcome data. Accordingly, the findings for more objective endpoints, particularly all‐cause mortality, are likely to be more robust than those for subjective outcomes such as QoL. Second, the evidence network was uneven, with limited direct head‐to‐head comparisons for some nodes, which increased reliance on indirect evidence and widened uncertainty for certain contrasts, particularly for less frequently studied strategies. Accordingly, some estimates for the primary outcomes remained imprecise, which limits definitive interpretation of several pairwise comparisons. This was reflected in the sensitivity analysis, in which the mortality findings largely preserved their direction, whereas comparisons involving AVNA_RV became nonestimable and the HF hospitalization findings were less stable. Formal subgroup analyses or meta‐regression were not performed because most network contrasts included too few studies to support stable estimates. Third, outcome characteristics likely constrained inference: CV mortality was infrequent and variably defined or adjudicated across trials, reducing power to detect between‐strategy differences, and QoL was assessed using different instruments, necessitating SMDs and limiting direct clinical interpretation of effect magnitude. In addition, because inconsistency was detected specifically in the QoL network, QoL‐related treatment rankings and pairwise estimates should be interpreted with greater caution than outcomes for which no important inconsistency was identified. Fourth, safety reporting was not standardized, and adverse events were inconsistently defined and collected across trials, precluding a granular comparison of procedural complications, device‐related harms, and drug toxicities alongside efficacy. In addition, although Egger′s regression tests were not statistically significant, visual asymmetry in the funnel plots and the limited number of studies in several networks mean that small‐study effects cannot be fully excluded.

## 5. Conclusion

In this NMA of RCTs in patients with HF and AF, AVNA_CRT and CA were associated with more favorable prognostic outcomes, with the most consistent and relatively robust signal observed for all‐cause mortality. AVNA_CRT also reduced HF hospitalization and worsening HF compared with AVNA_RV, indicating that the effectiveness of an AVNA strategy depends on pacing modality and supporting CRT as the preferred pacing approach when AVNA is chosen, although these comparisons should be interpreted with some caution because of network sparsity and limited precision. CA provided more consistent improvements in QoL and LVEF than medical strategies, but the QoL findings warrant cautious interpretation because of instrument heterogeneity and inconsistency within that network. Differences in CV mortality were not clearly distinguished across interventions. These findings support individualized selection of CA and AVNA_CRT according to clinical goals, patient phenotype, and procedural feasibility, and underscore the need for harmonized endpoints and longer follow‐up to refine treatment allocation.

## Author Contributions

C.D.: data curation, project administration, resources, investigation, supervision, writing review and editing. Y.X.: data curation, methodology, software, visualization, writing—original draft, writing—review and editing. C.F., Y.P., and J.H.: conceptualization, investigation, visualization, writing—original draft. B.L., H.L., and Z.W.: conceptualization, formal analysis, methodology, project administration, writing—review and editing. A.A.: formal analysis, methodology, visualization, writing—review and editing. T.W.: project administration, resources, supervision, validation, writing—original draft. C.D. and Y.X. have contributed to the work equally and should be regarded as co‐first authors.

## Funding

This study was supported by the Xinjiang Science and Technology, Tian Shan Ying cai Project (2024TSYCJU0008) and the Natural Science Foundation of Xinjiang Uygur Autonomous Region (10.13039/100009110, 2023D01C233).

## Ethics Statement

The study used previously published data, and institutional review board (IRB) approval was not required

## Consent

The authors have nothing to report.

## Conflicts of Interest

The authors declare no conflicts of interest.

## Supporting information


**Supporting Information** Additional supporting information can be found online in the Supporting Information section. Supporting Material 1: PRISMA checklist. Supporting Material 2: Search strategy. Supporting Material 3: Characteristics of studies and subjects included in the review. Supporting Material 4: Risk of Bias. Supporting Material 5: Publication bias, Supporting Material 6: Forest plot. Supporting Material 7: The results of node‐splitting. Supporting Material 8: Sensitivity analysis excluding studies with some concerns.

## Data Availability

The data that support the findings of this study are available from the corresponding author upon reasonable request. In line with the Wiley “Share Upon Reasonable Request” policy, deidentified data and relevant materials will be made available to qualified researchers upon reasonable request. Certain restrictions (e.g., ethical, privacy, or security concerns) may apply.
