# Supplementary material for: Comparative Effectiveness and Safety of Interventions for Atrial Fibrillation in Heart Failure: A Network Meta‐Analysis of Randomized Trials
Source: Cardiovasc Ther. 2026 Jul 7;2026:6035178. doi: 10.1155/cdr/6035178 (PMC13338893; doi:10.1155/cdr/6035178)
Supplement: Supplementary file 1 — Supporting Information Additional supporting information can be found online in the Supporting Information section. Supporting Material 1: PRISMA checklist. Supporting Material 2: Search strategy. Supporting Material 3: Characteristics of studies and subjects included in the review. Supporting Material 4: Risk of Bias. Supporting Material 5: Publication bias, Supporting Material 6: Forest plot. Supporting Material 7: The results of node‐splitting. Supporting Material 8: Sensitivity analysis excluding studies with some concerns. [file CDR-2026-6035178-s001.docx]

**Supplementary**

Table of Contents:

[Supplementary 1: PRISMA Checklist 1](#_Toc534204563)

[Supplementary 2: Search Strategy 4](#_Toc1894445881)

[Supplementary 3: Characteristics of studies and subjects included in the review 6](#_Toc1347370016)

[Supplementary 4: Risk of Bias](#_Toc1262935083) 22

[Supplementary 5: Publication bias](#_Toc431254745) 24

[Supplementary 6: Forest plot](#_Toc1262935083) 27

[Supplementary 7: The results of node-splitting](#_Toc1262935083) 30

[Supplementary 8: Sensitivity analysis excluding studies with some concerns](#_Toc1262935083) 33

**Supplementary 1: PRISMA Checklist**

| Item # | Checklist Item | Reported on Page # |
| --- | --- | --- |
|  |  | 1 |
| 1 | Identify the report as a systematic review incorporating a network meta-analysis (or related form of meta-analysis). | 1 |
| 2 | Provide a structured summary including, as applicable:  **Background:** main objectives  **Methods:** data sources; study eligibility criteria, participants, and interventions; study appraisal; and *synthesis methods, such as network meta-analysis.*  **Results:** number of studies and participants identified; summary estimates with corresponding confidence/credible intervals; treatment rankings may also be discussed. Authors may choose to summarize pairwise comparisons against a chosen treatment included in their analyses for brevity.  **Discussion/Conclusions:** limitations; conclusions and implications of findings.  **Other:** primary source of funding; systematic review registration number with registry name. | 1-2 |
| 3 | Describe the rationale for the review in the context of what is already known*, including mention of why a meta-analysis of randomized controlled trials and dose-response analysis has been conducted.* | 3-5 |
| 4 | Provide an explicit statement of questions being addressed, with reference to participants, interventions, comparisons, outcomes, and study design (PICOS). | 5-6 |
| 5 | Indicate whether a review protocol exists and if and where it can be accessed (e.g., Web address); and, if available, provide registration information, including registration number. | 5 |
| 6 | Specify study characteristics (e.g., PICOS, length of follow-up) and report characteristics (e.g., years considered, language, publication status) used as criteria for eligibility, giving rationale. *Clearly describe eligible treatments included in the treatment network, and note whether any have been clustered or merged into the same node (with justification).* | 28-29 |
| 7 | Describe all information sources (e.g., databases with dates of coverage, contact with study authors to identify additional studies) in the search and date last searched. | 5 |
| 8 | Present full electronic search strategy for at least one database, including any limits used, such that it could be repeated. | Supplementary 2 |
| 9 | State the process for selecting studies (i.e., screening, eligibility, included in systematic review, and, if applicable, included in the meta-analysis). | 5-6 |
| 10 | Describe method of data extraction from reports (e.g., piloted forms, independently, in duplicate) and any processes for obtaining and confirming data from investigators. | 6-7 |
| 11 | List and define all variables for which data were sought (e.g., PICOS, funding sources) and any assumptions and simplifications made. | 7-8 |
| S1 | Describe methods used to explore the geometry of the treatment network under study and potential biases related to it. This should include how the evidence base has been graphically summarized for presentation, and what characteristics were compiled and used to describe the evidence base to readers. | 8 |
| 12 | Describe methods used for assessing risk of bias of individual studies (including specification of whether this was done at the study or outcome level), and how this information is to be used in any data synthesis. | 8, Supplementary 4 |
| 13 | State the principal summary measures (e.g., risk ratio, difference in means). Also describe the use of additional summary measures assessed, such as treatment rankings and surface under the cumulative ranking curve (SUCRA) values, as well as modified approaches used to present summary findings from meta-analyses. | 8-10 |
| 14 | Describe the methods of handling data and combining results of studies for each network meta-analysis. This should include, but not be limited to:   - Handling of multi-arm trials; - Selection of variance structure; - Selection of prior distributions in Bayesian analyses; and - Assessment of model fit. | 8-10 |
| S2 | Describe the statistical methods used to evaluate the agreement of direct and indirect evidence in the treatment network(s) studied. Describe efforts taken to address its presence when found. | Supplementary 7 |
| 15 | Specify any assessment of risk of bias that may affect the cumulative evidence (e.g., publication bias, selective reporting within studies). | 9,  Supplementary 6 |
| 16 | Describe methods of additional analyses if done, indicating which were pre-specified. This may include, but not be limited to, the following:   - Sensitivity or subgroup analyses; - Meta-regression analyses; - Alternative formulations of the treatment network; and - Use of alternative prior distributions for Bayesian analyses (if applicable). | 8-10 |
| 17 | Give numbers of studies screened, assessed for eligibility, and included in the review, with reasons for exclusions at each stage, ideally with a flow diagram. | Figure 1 |
| S3 | Provide a network graph of the included studies to enable visualization of the geometry of the treatment network. | Figure 5 |
| S4 | Provide a brief overview of characteristics of the treatment network. This may include commentary on the abundance of trials and randomized patients for the different interventions and pairwise comparisons in the network, gaps of evidence in the treatment network, and potential biases reflected by the network structure. | Supplementary 7 |
| 18 | For each study, present characteristics for which data were extracted (e.g., study size, PICOS, follow-up period) and provide the citations. | Supplementary 3 |
| 19 | Present data on risk of bias of each study and, if available, any outcome level assessment. | 11,  Supplementary 4, and Supplementary 6 |
| 20 | For all outcomes considered (benefits or harms), present, for each study: 1) simple summary data for each intervention group, and 2) effect estimates and confidence intervals. *Modified approaches may be needed to deal with information from larger networks.* | Supplementary 3 |
| 21 | Present results of each meta-analysis done, including confidence/credible intervals. In larger networks, authors may focus on comparisons versus a particular comparator (e.g. placebo or standard care), with full findings presented in an appendix. League tables and forest plots may be considered to summarize pairwise comparisons. If additional summary measures were explored (such as treatment rankings), these should also be presented. | Figure 2, Figure 3, and Figure 4 |
| S5 | Describe results from investigations of inconsistency. This may include such information as measures of model fit to compare consistency and inconsistency models, *P* values from statistical tests, or summary of inconsistency estimates from different parts of the treatment network. | Supplementary 7, and Supplementary 8 |
| 22 | Present results of any assessment of risk of bias across studies for the evidence base being studied. | 11,  Supplementary 4, and Supplementary 6 |
| 23 | Give results of additional analyses, if done (e.g., sensitivity or subgroup analyses, meta-regression analyses*, alternative network geometries studied, alternative choice of prior distributions for Bayesian analyses,* and so forth). | Figure 4,  and Figure 5 |
| 24 | Summarize the main findings, including the strength of evidence for each main outcome; consider their relevance to key groups (e.g., healthcare providers, users, and policy-makers). | 14-19 |
| 25 | Discuss limitations at study and outcome level (e.g., risk of bias), and at review level (e.g., incomplete retrieval of identified research, reporting bias). *Comment on the validity of the assumptions, such as transitivity and consistency. Comment on any concerns regarding network geometry (e.g., avoidance of certain comparisons).* | 19 |
| 26 | Provide a general interpretation of the results in the context of other evidence, and implications for future research. | 19 |
| 27 | Describe sources of funding for the systematic review and other support (e.g., supply of data); role of funders for the systematic review. This should also include information regarding whether funding has been received from manufacturers of treatments in the network and/or whether some of the authors are content experts with professional conflicts of interest that could affect use of treatments in the network. | 19 |

PICOS = population, intervention, comparators, outcomes, study design.

# Supplementary 2: Search Strategy

***Search Strategy:***

| #9 | Search: (((("Atrial Fibrillation"[Mesh] OR "atrial fibrillation"[tiab] OR "AF"[tiab] OR "AFib"[tiab])) AND (("Heart Failure"[Mesh] OR "Heart Failure, Systolic"[Mesh] OR "Heart Failure, Diastolic"[Mesh] OR "Cardiomyopathy, Dilated"[Mesh] OR "heart failure"[tiab] OR "cardiac failure"[tiab] OR "congestive heart failure"[tiab] OR "left ventricular dysfunction"[tiab] OR "LV dysfunction"[tiab] OR HFrEF[tiab] OR HFpEF[tiab] OR HFmrEF[tiab]))) AND ((("Catheter Ablation"[Mesh] OR "catheter ablation"[tiab] OR "pulmonary vein isolation"[tiab] OR "PVI"[tiab] OR "pulmonary veins"[Mesh] OR "radiofrequency ablation"[tiab] OR "RF ablation"[tiab] OR "cryoballoon"[tiab] OR "cryoablation"[tiab] OR "pulsed field ablation"[tiab] OR "PFA"[tiab]) OR ("Maze procedure"[Mesh] OR "Cox maze"[tiab] OR maze[tiab] OR "surgical ablation"[tiab])) OR ("atrioventricular node"[Mesh] OR "AV node ablation"[tiab] OR "AV nodal ablation"[tiab] OR "ablate and pace"[tiab] OR "pace and ablate"[tiab] OR "cardiac resynchronization therapy"[Mesh] OR "cardiac resynchronization"[tiab] OR "CRT"[tiab] OR "biventricular pacing"[tiab]))) AND ((randomized controlled trial[pt] OR controlled clinical trial[pt] OR randomized[tiab] OR placebo[tiab] OR drug therapy[sh] OR randomly[tiab] OR trial[tiab] OR groups[tiab]) NOT (animals[mh] NOT humans[mh])) |
| --- | --- |
| #8 | Search: (("Catheter Ablation"[Mesh] OR "catheter ablation"[tiab] OR "pulmonary vein isolation"[tiab] OR "PVI"[tiab] OR "pulmonary veins"[Mesh] OR "radiofrequency ablation"[tiab] OR "RF ablation"[tiab] OR "cryoballoon"[tiab] OR "cryoablation"[tiab] OR "pulsed field ablation"[tiab] OR "PFA"[tiab]) OR ("Maze procedure"[Mesh] OR "Cox maze"[tiab] OR maze[tiab] OR "surgical ablation"[tiab])) OR ("atrioventricular node"[Mesh] OR "AV node ablation"[tiab] OR "AV nodal ablation"[tiab] OR "ablate and pace"[tiab] OR "pace and ablate"[tiab] OR "cardiac resynchronization therapy"[Mesh] OR "cardiac resynchronization"[tiab] OR "CRT"[tiab] OR "biventricular pacing"[tiab]) |
| #7 | Search: (randomized controlled trial[pt] OR controlled clinical trial[pt] OR randomized[tiab] OR placebo[tiab] OR drug therapy[sh] OR randomly[tiab] OR trial[tiab] OR groups[tiab]) NOT (animals[mh] NOT humans[mh]) |
| #6 | Search: "atrioventricular node"[Mesh] OR "AV node ablation"[tiab] OR "AV nodal ablation"[tiab] OR "ablate and pace"[tiab] OR "pace and ablate"[tiab] OR "cardiac resynchronization therapy"[Mesh] OR "cardiac resynchronization"[tiab] OR "CRT"[tiab] OR "biventricular pacing"[tiab] |
| #5 | Search: "Maze procedure"[Mesh] OR "Cox maze"[tiab] OR maze[tiab] OR "surgical ablation"[tiab] |
| #4 | Search: "Catheter Ablation"[Mesh] OR "catheter ablation"[tiab] OR "pulmonary vein isolation"[tiab] OR "PVI"[tiab] OR "pulmonary veins"[Mesh] OR "radiofrequency ablation"[tiab] OR "RF ablation"[tiab] OR "cryoballoon"[tiab] OR "cryoablation"[tiab] OR "pulsed field ablation"[tiab] OR "PFA"[tiab] |
| #3 | Search: (("Atrial Fibrillation"[Mesh] OR "atrial fibrillation"[tiab] OR "AF"[tiab] OR "AFib"[tiab])) AND (("Heart Failure"[Mesh] OR "Heart Failure, Systolic"[Mesh] OR "Heart Failure, Diastolic"[Mesh] OR "Cardiomyopathy, Dilated"[Mesh] OR "heart failure"[tiab] OR "cardiac failure"[tiab] OR "congestive heart failure"[tiab] OR "left ventricular dysfunction"[tiab] OR "LV dysfunction"[tiab] OR HFrEF[tiab] OR HFpEF[tiab] OR HFmrEF[tiab])) |
| #2 | Search: ("Heart Failure"[Mesh] OR "Heart Failure, Systolic"[Mesh] OR "Heart Failure, Diastolic"[Mesh] OR "Cardiomyopathy, Dilated"[Mesh] OR "heart failure"[tiab] OR "cardiac failure"[tiab] OR "congestive heart failure"[tiab] OR "left ventricular dysfunction"[tiab] OR "LV dysfunction"[tiab] OR HFrEF[tiab] OR HFpEF[tiab] OR HFmrEF[tiab]) |
| #1 | Search: ("Atrial Fibrillation"[Mesh] OR "atrial fibrillation"[tiab] OR "AF"[tiab] OR "AFib"[tiab]) |

# Supplementary 3: Characteristics of studies and subjects included in the review

| **Study** | **Country/Region** | **Study design** | **Subjects**  **(intervention/ control)** | **Sex (male/female)**  **(intervention/ control)** | **Mean age**  **(intervention/ control)** | **LVEF**  **(%)** | **AF type** | **AF duration** | **Intervention detail** | | | | **Follow-up** | **Outcomes** |
| --- | --- | --- | --- | --- | --- | --- | --- | --- | --- | --- | --- | --- | --- | --- |
|  |  |  |  |  |  |  |  |  | **Intervention group** | | **Control group** | |  |  |
| Brignole et al. (1998) | Italy | Open-label RCT | 66 (32/34) | 18/14 vs. 13/21 | 72 ± 9 vs 72 ± 9 | 44 ± 11 vs. 44 ± 15 | Chronic AF ≥ 6 months | 5.7±6.9 vs. 4.1±5 years | AVNA_RV | AV node ablation (target: complete and permanent AV block) + VVIR single-chamber pacing (lower limit 80 bpm, upper limit 120 bpm) | CON | The drug treatment group used traditional HF medications (ACE inhibitors, diuretics, nitrates, digitalis) + rate-controlling drugs (calcium channel blockers, beta-blockers, amiodarone, sotalol). |  | LVEF, QoL, All-cause mortality, Cardiovascular mortality, Worsening heart failure |
| Brignole et al. (2011) | Italy, Spain, Greece | Single-blind RCT | 186 (97 / 89) | 65 / 32 vs. 65 / 24 | 72 ± 9 vs 72 ± 8 | 41.0 ± 12.0 vs. 40.0 ± 12.0 | Permanent AF | 24 (12–48) vs. 17 (10–40) months | AVNA_CRT | Patients underwent atrioventricular junction ablation followed by cardiac resynchronization therapy (CRT) with optimized biventricular pacing. | AVNA_RV | Patients underwent atrioventricular junction ablation followed by conventional right ventricular apical pacing. | 20 months (IQR 11–24) | LVEF, All-cause mortality, Worsening heart failure, Heart failure hospitalization |
| Brignole et al. (2018) | Italy, Spain, Russia | Open-label RCT | 102 (50 / 52) | 28 / 22 vs. 28 / 24 | 71 ± 12 vs. 72 ± 9 | 41 ± 12 vs. 40 ± 12 | Permanent AF（>6 months） | 13 (8–36) vs. 18 (8–43) months | AVNA_CRT | Atrioventricular junction ablation followed by cardiac resynchronization therapy (CRT). | MRC | Optimal pharmacological rate-control therapy. | 16 months | QoL, All-cause mortality, Worsening heart failure Heart failure hospitalization |
| Brignole et al. (2021) | Italy, Spain, Netherlands, France | Open-label RCT | 133 (63 / 70) | 35 / 28 vs. 36 / 34 | 72 ± 11 vs. 74 ± 9 | 41 ± 12 vs. 41 ± 12 | Permanent AF（>6 months） | 19 (8–48) vs. 18 (8–38) months | AVNA_CRT | Atrioventricular junction ablation followed by cardiac resynchronization therapy (CRT). | MRC | Optimal pharmacological rate-control therapy. | 29 months (range 1–56) | All-cause mortality Heart failure hospitalization |
| Di Biase et al. (2016) | Multinational | Open-label RCT | 203 (102 / 101) | 77 / 25 vs. 74 / 27 | 62 ± 10 vs. 60 ± 11 | 31 ± 7 vs. 32 ± 6 | Persistent AF | 8.6 ± 3.2 vs. 8.4 ± 4.1 months | CA | Patients underwent catheter ablation for atrial fibrillation, with pulmonary vein isolation as the mandatory lesion set, with additional left atrial posterior wall isolation at the operator’s discretion. | RDC | Patients received amiodarone-based medical therapy for rhythm control, in addition to guideline-directed heart failure treatment. | ≥24 months | LVEF, QoL, All-cause mortality, Cardiovascular mortality Heart failure hospitalization |
| Doshi et al. (2005) | USA & Canada | Single-blind RCT | 184 (103 / 81) | 65 / 38 vs. 52 / 29 | 70 ± 10 vs. 67 ± 10 | 47 ± 16 vs. 45 ± 15 | Chronic AF (>30 days) | NR | AVNA_CRT | Atrioventricular node ablation followed by biventricular (CRT) pacing. | AVNA_RV | Atrioventricular node ablation followed by conventional right ventricular apical pacing. | 21 ± 8 months | LVEF, All-cause mortality, Cardiovascular mortality |
| Huang et al. (2022) | United Kingdom | Single-blind RCT | 50 (26 / 24) | 25 / 1 vs. 23 / 1 | 55 ± 12 vs. 60 ± 10 | 31.8 ± 7.7 vs. 33.7 ± 12.1 | Persistent AF / Long-standing persistent AF | 24 (17–33) vs. 24 (12–48) | CA | Catheter ablation for atrial fibrillation with pulmonary vein isolation as the cornerstone, supplemented by linear lesions and complex fractionated atrial electrogram ablation when required. | MRC | Optimized medical rate-control therapy according to contemporary heart failure and atrial fibrillation guidelines. | 12 months | LVEF, QoL, All-cause mortality, Cardiovascular mortality |
| Jones et al. (2013) | United Kingdom | Open-label RCT | 52 (26 / 26) | 21/5 vs. 24/2 | 64 ± 10 vs. 62 ± 9 | 22 ± 8 vs. 25 ± 7 | Persistent AF | 23 ± 22 vs. 24 ± 29 months | CA | Catheter ablation for persistent atrial fibrillation using a stepwise strategy with pulmonary vein isolation as the cornerstone, supplemented by linear ablation and complex fractionated atrial electrogram ablation when required. | MRC | Optimized pharmacological rate-control therapy with beta-blockers and/or digoxin according to guideline-defined heart-rate targets. | 12 months | LVEF, QoL, All-cause mortality |
| Khan et al. (2008) | Multinational | Open-label RCT | 81 (41 / 40) | 39 / 2 vs. 35 / 5 | 60 ± 8 vs. 61 ± 8 | 27 ± 8 vs. 29 ± 7 | Paroxysmal AF：49%（PVI） vs 54%（AVNA + BiV） Persistent / long-standing persistent AF：51% vs 46% | 4.0 ± 2.4 vs. 3.9 ± 2.8 years | CA | Pulmonary vein isolation for atrial fibrillation using circumferential antral ablation, with additional linear or complex fractionated atrial electrogram ablation at operator discretion. | AVNA_CRT | Atrioventricular node ablation followed by implantation of a biventricular ICD with cardiac resynchronization pacing. | 6 months | LVEF, QoL, All-cause mortality, Cardiovascular mortality |
| Kuck et al. (2019) | Multinational | Open-label RCT | 140 (68 / 72) | 60 / 8 vs. 66 / 6 | 65 ± 8 vs. 65 ± 8 | 27.8 ± 9.5 vs. 24.8 ± 8.8 | Persistent AF：81%（Ablation） vs 72%（BMT） Long-standing persistent AF：19% vs 28% | Baseline rhythm AF：82%（Ablation） vs 92%（BMT） | CA | Catheter ablation of atrial fibrillation with mandatory pulmonary vein isolation, supplemented by linear lesions and/or complex fractionated atrial electrogram ablation at operator discretion. | CON | Best medical therapy including rate or rhythm control according to contemporary atrial fibrillation and heart failure guidelines | 358 ± 71 days | LVEF, QoL, All-cause mortality, Cardiovascular mortality, Worsening heart failure |
| MacDonald et al. (2011) | United Kingdom | Open-label RCT | 41 (22 / 19) | 17 / 5 vs. 15 / 4 | 62.3 ± 6.7 vs. 64.4 ± 8.3 | 16.1 ± 7.1 vs. 16.1 ± 7.1 | Persistent AF | 44 ± 36.5 vs. 44 ± 36.5 months | CA | Catheter radiofrequency ablation for persistent atrial fibrillation with mandatory pulmonary vein isolation, supplemented by linear lesions and complex fractionated atrial electrogram ablation; repeat ablation permitted if AF recurred. | MRC | Continued optimal medical therapy with a rate-control strategy according to heart failure guidelines. | 9.7 ± 2.7 vs. 6.9 ± 0.9 months | LVEF, QoL, All-cause mortality, Cardiovascular mortality |
| Marrouche et al. (2018) | Multinational | Open-label RCT | 363 (179 / 184) | 156 / 23 vs. 155 / 29 | 64 ± 3.8 vs. 64 ± 4.4 | 31.8 ± 9.6 vs. 31.8 ± 9.6 | Paroxysmal AF：30% vs 35% Persistent AF：70% vs 65% Long-standing persistent AF (>1 year)：28% vs 30% | NR | CA | Catheter ablation for atrial fibrillation with mandatory pulmonary vein isolation and optional additional lesions at operator discretion; repeat ablation permitted for recurrence. | CON | Guideline-directed medical therapy for atrial fibrillation using rate or rhythm control strategies, without mandated antiarrhythmic drug selection. | 37.8 months | LVEF, All-cause mortality, Cardiovascular mortality Heart failure hospitalization |
| Moersdorf et al. (2024) | Multinational | Open-label RCT | 194 (97 / 97) | 86 / 11 vs. 71 / 26 | 62 ± 11 vs. 65 ± 11 | 29 ± 6 vs. 29 ± 6 | Paroxysmal AF：25 (26%) / 34 (35%) Persistent AF：59 (61%) / 49 (51%) Long-standing persistent AF：13 (13%) / 14 (14%) | 3 ± 5 vs. 4 ± 4 years | CA | Catheter ablation of atrial fibrillation in addition to guideline-directed medical therapy, with continuous rhythm monitoring via implanted cardiac devices. | CON | Guideline-directed medical therapy alone for atrial fibrillation and heart failure, without catheter ablation. | 18.0 months | LVEF, All-cause mortality, Cardiovascular mortality |
| Parkash et al. (2022) | Multinational | Open-label RCT | 411 (214 / 197) | 157 / 57 vs. 148 / 49 | 65.9 ± 8.6 vs. 67.5 ± 8.0 | NR | NR | 19.2 ± 21.5 vs. 23.0 ± 31.1 | CA | Catheter ablation–based rhythm control for atrial fibrillation, with pulmonary vein isolation as the mandatory lesion set and additional ablation at the operator’s discretion, plus guideline-directed heart failure therapy. | MRC | Rate-control strategy using pharmacological therapy, with atrioventricular node ablation and pacing permitted when rate control was inadequate, in addition to guideline-directed heart failure therapy. | 37.4 months | LVEF, QoL, All-cause mortality, Worsening heart failure |
| Song et al. (2025) | China | Open-label RCT | 89 (45/44) | 28/17 vs. 32/12 | 69.5 ± 3.8 vs. 69.5 ± 4.1 | 36.1 ± 2.7 vs. 36.5 ± 2.1 | 100% Persistent Atrial Fibrillation (Persistent AF) | 24.0 ± 22.6 vs. 31.0 ± 20.6 months | CA | Patients underwent radiofrequency catheter ablation, including pulmonary vein isolation and additional linear ablation of the left atrium. Post-procedure, oral anticoagulants were prescribed for 3 months, and antiarrhythmic drugs (AADs) were discontinued unless AF recurred during the 3-month blanking period. | CON | Patients received medical therapy aimed at controlling heart rate (60–80 bpm) using standard medications, including ACE inhibitors, beta-blockers, and SGLT2 inhibitors. Antiarrhythmic drugs were used for rate control when necessary, with follow-up assessments via Holter monitoring. | 12 months | LVEF, Heart failure hospitalization |
| Sugumar et al. (2020) | Australia | Open-label RCT | 66 (33/33) | 30/3 vs. 28/5 | 62 ± 10.2 vs. 61.5 ± 7.2 | 32.8 ± 8.8 vs. 33.2 ± 9.0 | 100% Persistent Atrial Fibrillation (Persistent AF) | 2.3 ± 2.3 years vs. 2.4 ± 2.5 years | CA | Catheter ablation was performed under general anesthesia, with pulmonary vein isolation (100% success) and posterior wall isolation in 94.1% of patients. The procedure was guided by 3D mapping systems and radiofrequency ablation was applied (25-30 W). Anticoagulation was maintained throughout the procedure, and antiarrhythmic drugs were discontinued after a 3-month blanking period. Repeat procedures were offered for arrhythmia recurrence, with a mean of 1.4 procedures per patient. | MRC | Patients in the MRC group received medical therapy to control heart rate, with a target resting heart rate <80 bpm and a 24-hour ventricular rate <100 bpm. The treatment was based on clinical guidelines, including beta-blockers, ACE inhibitors, and diuretics. Follow-up included 24-hour Holter monitoring at 3 and 6 months to assess heart rate control. | 4.0 ± 0.9 years | LVEF, All-cause mortality |
| Zakeri et al. (2023) | United Kingdom | Open-label RCT | 102 (52/50) | 46/6 vs. 47/3 | 60 ± 11 vs. 63 ± 9 | 31 ± 11 vs. 33 ± 10 | 100% Persistent Atrial Fibrillation (Persistent AF) | 36 (IQR: 12–66) vs. 21 (IQR: 9–60) months | CA | Radiofrequency catheter ablation with pulmonary vein isolation (PVI) and additional linear or complex fractionated electrogram (CFAE) ablation based on operator’s discretion. | MRC | Pharmacological rate control (β-blockers, digoxin) with target heart rate ≤80 bpm at rest and ≤110 bpm after exertion. | 7.8 years (IQR: 3.9–9.9 years). | LVEF, All-cause mortality |
| Hagens et al. (2005) | Netherlands | Open-label RCT | 261 (131/130) | 85/46 vs. 85/45 | 69±8 vs 69±9 | NR | 100% Persistent Atrial Fibrillation (Persistent AF) | 499 days vs. 562 days | RDC | Rhythm control: serial electrical cardioversion + antiarrhythmic drugs (sotalol / class IC / amiodarone). | MRC | Negative chronotropic drugs (digitalis, beta-blockers, non-dihydropyridine calcium channel blockers) | 2.3±0.6 years | QoL, All-cause mortality, Cardiovascular mortality, Worsening heart failure Heart failure hospitalization |
| Ökcün et al. (2004) | Turkey | Open-label RCT | 154 (70/84) | 46/24 vs. 57/27 | 61±10 vs. 58±12 | 31±8 vs 33±15 | 100% Persistent Atrial Fibrillation (Persistent AF) | 11±7 vs 13±6 months | RDC | After excluding thrombus via transesophageal echocardiography, sinus rhythm was restored using amiodarone (intravenous + oral) or electrical cardioversion. Maintenance of sinus rhythm: amiodarone 200 mg four times daily Anticoagulation: Warfarin was discontinued one month after cardioversion. | MRC | Medication to control ventricular rate: Digoxin + Metoprolol (target resting heart rate < 80 bpm) Continuous anticoagulation: Warfarin INR 2.0–3.0 Basic treatment: ACE inhibitors/ARBs, beta-blockers, etc. | 35±21 vs 37±19 months | LVEF, All-cause mortality, Cardiovascular mortality |
| Packer et al. (2021) | USA | Open-label RCT | 778 (378 / 400) | 207/171 vs. 226/174 | 67.7 ± 8.2 vs 67.3 ± 8.2 | 55.0 ± 7.4 vs 56.0 ± 8.9 | Paroxysmal：110/378 (29.1%) vs 136/400 (34.0%)  Persistent：221/378 (58.5%) vs 209/400 (52.3%)  Longstanding persistent：47/378 (12.4%) vs 55/400 (13.8%) | 1.1 ± 2.3 years vs. 1.2 ± 2.1 years | CA | Catheter ablation with pulmonary vein isolation as the cornerstone (with additional lesion sets at operator discretion), delivered on top of guideline-directed background therapy, with antiarrhythmic drugs allowed as clinically indicated. | CON | Conventional medical therapy using rate- and/or rhythm-control drugs per guideline-based management (antiarrhythmic drugs permitted), with background heart-failure therapy continued. | 48.5 months | QoL, All-cause mortality, Cardiovascular mortality Heart failure hospitalization |
| Prabhu et al. (2017) | Australia | Open-label RCT | 66 (33/33) | 31/2 vs. 29/4 | 59±11 vs 62±9.4 | 32±9.4 vs 34±7.8 | 100% Persistent Atrial Fibrillation (Persistent AF) | 23±18 vs 21±15 months | CA | Catheter ablation using wide antral pulmonary vein isolation plus left atrial posterior wall isolation (roof and inferior lines), performed under general anesthesia with protocolized peri-procedural anticoagulation; antiarrhythmic drugs were stopped pre-procedure (except amiodarone) and subsequent rhythm follow-up was supported by implantable loop recording. | MRC | Guideline-based pharmacologic rate control with titration to prespecified heart-rate targets (resting <80 bpm, 24-h mean <100 bpm, post-6MWT <110 bpm), with serial Holter monitoring to guide dose adjustment; patients remained in AF without attempts at rhythm restoration. | 6 months | LVEF, QoL |
| Roy et al. (2008) | Canada | Open-label RCT | 1376 (682 / 694) | 532 / 150 vs. 590 / 104 | 66 ± 11 vs 67 ± 11 | 27 ± 6 vs 27 ± 6 | Paroxysmal 33% vs 30%  Persistent 67% vs 70% | AF ≥6 months: 41% vs 46% | RDC | A strategy aimed at restoring and maintaining sinus rhythm using antiarrhythmic drug therapy (amiodarone as first-line, with alternatives as needed) plus protocolized direct-current cardioversion when indicated, on top of guideline-directed heart-failure management and anticoagulation. | MRC | A strategy focused on ventricular rate control using rate-slowing drugs (e.g., beta-blockers ± digoxin, with other agents as required) to prespecified heart-rate targets, with atrioventricular node ablation and permanent pacing permitted if pharmacologic control was inadequate, alongside guideline-directed heart-failure therapy and anticoagulation. | 37 ± 19 months | All-cause mortality, Cardiovascular mortality, Worsening heart failure Heart failure hospitalization |
| Shelton et al. (2009) | UK | Open-label RCT | 61 (30/31) | 26/4 vs. 25/6 | 72.0±5.4 vs 72.7±8.3 | NR | 100% Persistent Atrial Fibrillation (Persistent AF) | 14±13 vs 15±12 months | RDC | Amiodarone-based rhythm control with planned electrical cardioversion as needed to restore and maintain sinus rhythm, with anticoagulation continued. | MRC | Pharmacologic rate control using beta-blockers and/or digoxin to achieve prespecified ventricular rate targets without attempts at rhythm restoration, with anticoagulation continued. | 12 months | QoL, All-cause mortality, Cardiovascular mortality |

NR, not reported; RCT, randomized controlled trial; HF, heart failure; AF, atrial fibrillation; LVEF, left ventricular ejection fraction; NYHA, New York Heart Association; CA, catheter ablation; PVI, pulmonary vein isolation; AVNA, atrioventricular node ablation; CRT, cardiac resynchronization therapy; RV, right ventricle; AVNA_CRT, AVNA plus CRT pacing; AVNA_RV, AVNA plus right ventricular pacing; MRC, medical rate control; RDC, rhythm drug control; AAD, antiarrhythmic drug; CON, conventional care; QoL, quality of life; 6MWT, 6-minute walk test; CV, cardiovascular. Missing baseline descriptive data were recorded as NR, and no formal imputation was performed.

# Supplementary 4: Risk of Bias

| **Study** | Randomization process | Deviations from intended interventions | Missing outcome data | Measurement of the outcome | Selection of the reported result | Overall Bias |
| --- | --- | --- | --- | --- | --- | --- |
| Brignole et al. (1998) | Some concerns | Some concerns | Some concerns | Low | Low | Some concerns |
| Brignole et al. (2011) | Low | Some concerns | Low | Low | Low | Some concerns |
| Brignole et al. (2018) | Low | Low | Low | Low | Low | Low |
| Brignole et al. (2021) | Low | Low | Low | Low | Low | Low |
| Di Biase et al. (2016) | Low | Low | Low | Low | Low | Low |
| Doshi et al. (2005) | Low | Low | Some concerns | Low | Low | Some concerns |
| Huang et al. (2022) | Low | Low | Low | Low | Low | Low |
| Jones et al. (2013) | Low | Some concerns | Low | Low | Low | Some concerns |
| Khan et al. (2008) | Low | Low | Low | Low | Low | Low |
| Kuck et al. (2019) | Low | Some concerns | Some concerns | Low | Low | Some concerns |
| MacDonald et al. (2011) | Low | Low | Low | Low | Low | Low |
| Marrouche et al. (2018) | Low | Low | Low | Low | Low | Low |
| Moersdorf et al. (2024) | Low | Low | Some concerns | Low | Low | Some concerns |
| Parkash et al. (2022) | Low | Low | Low | Low | Low | Low |
| Song et al. (2025) | Low | Low | Low | Low | Low | Low |
| Sugumar et al. (2020) | Low | Low | Low | Low | Low | Low |
| Zakeri et al. (2023) | Low | Low | Low | Low | Low | Low |
| Hagens et al. (2005) | Low | Low | Some concerns | Low | Low | Some concerns |
| Ökcün et al. (2004) | Low | Low | Low | Low | Low | Low |
| Packer et al. (2021) | Some concerns | Low | Some concerns | Low | Low | Some concerns |
| Prabhu et al. (2017) | Low | Low | Some concerns | Low | Low | Some concerns |
| Roy et al. (2008) | Low | Low | Low | Low | Low | Low |
| Shelton et al. (2009) | Low | Low | Low | Low | Low | Low |

# Supplementary 5: Publication bias


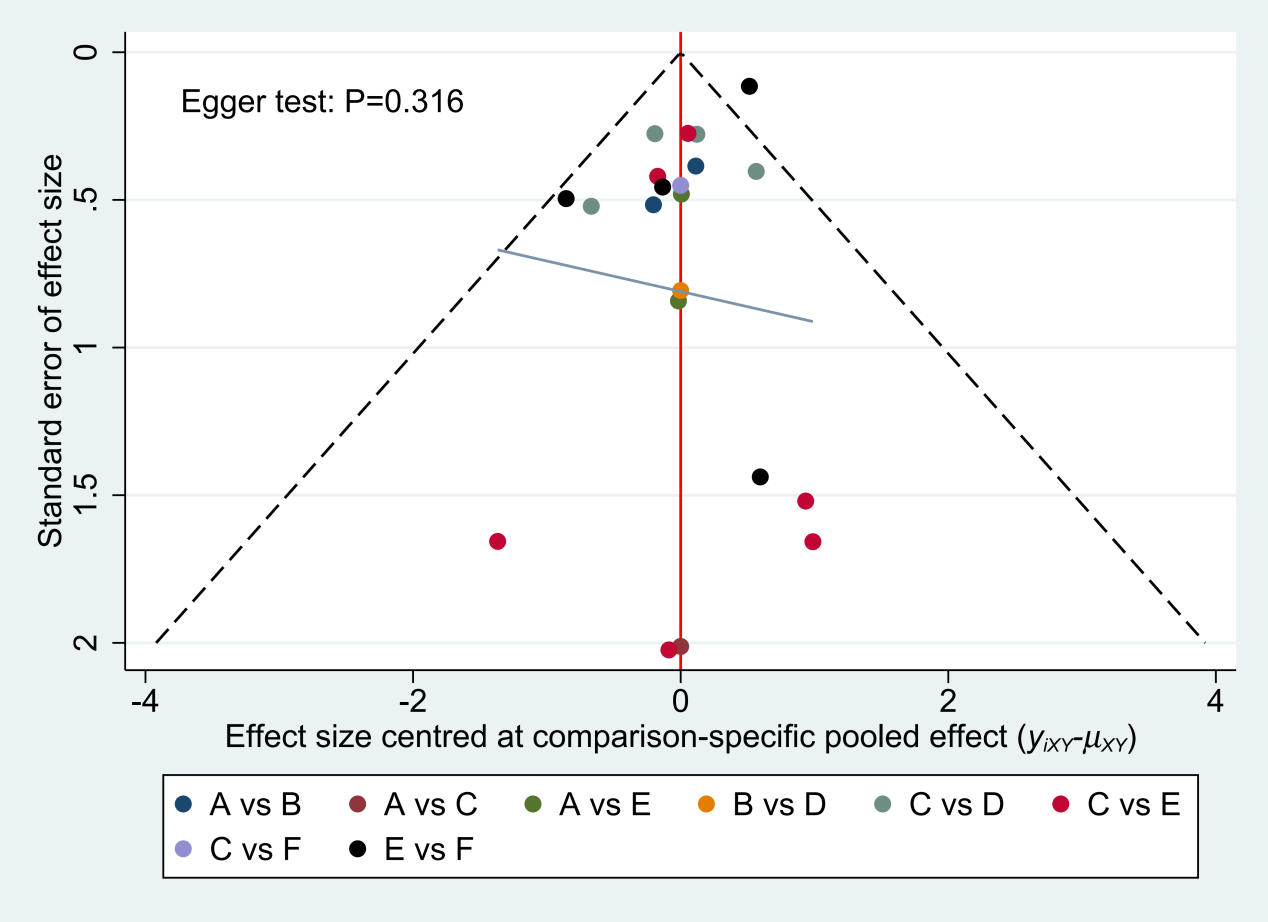


Figure 5.1 The funnel plot of All-cause mortality. The result of Egger test showed the p=0.316.


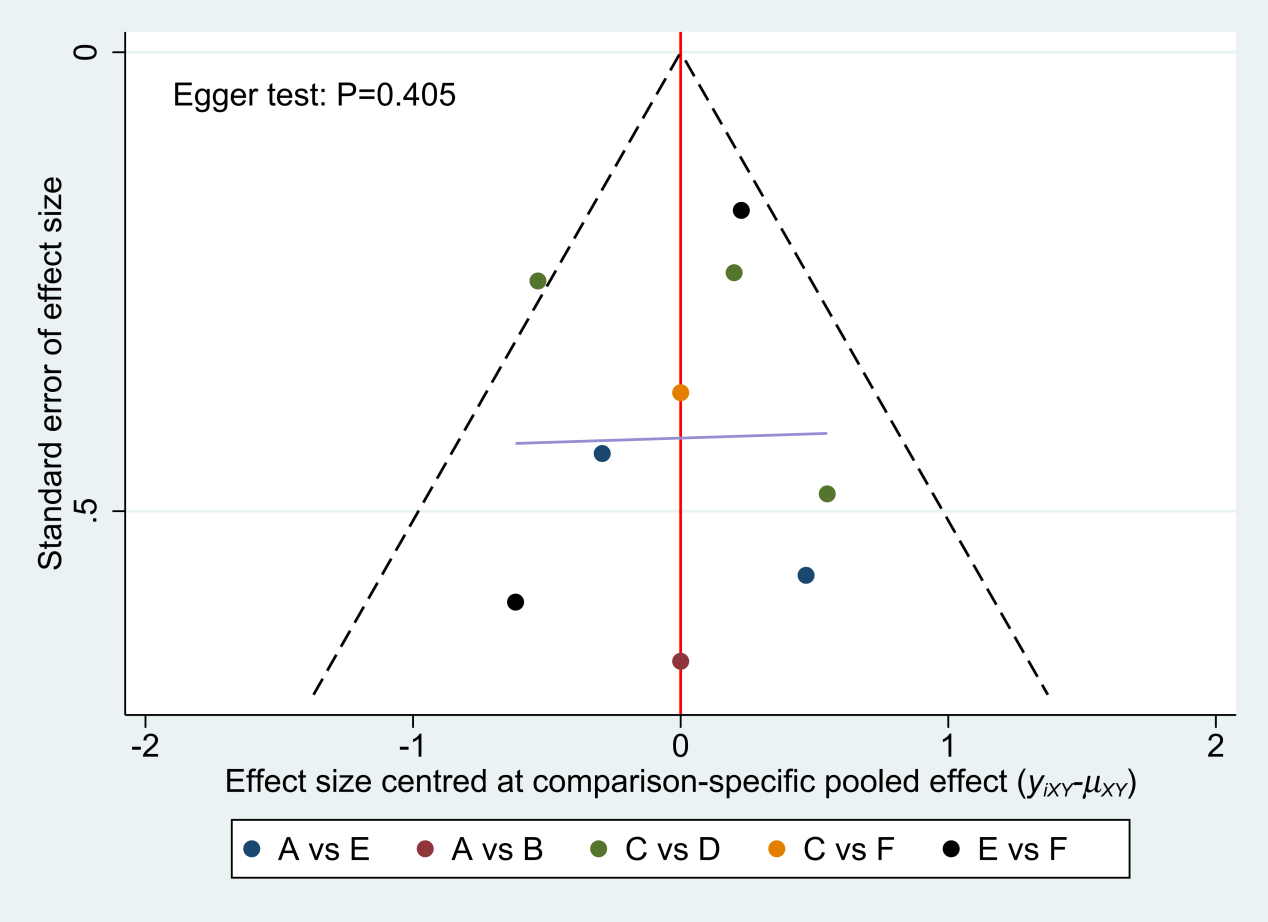


Figure 5.2 The funnel plot of Heart failure hospitalization. The result of Egger test showed the p=0.405.


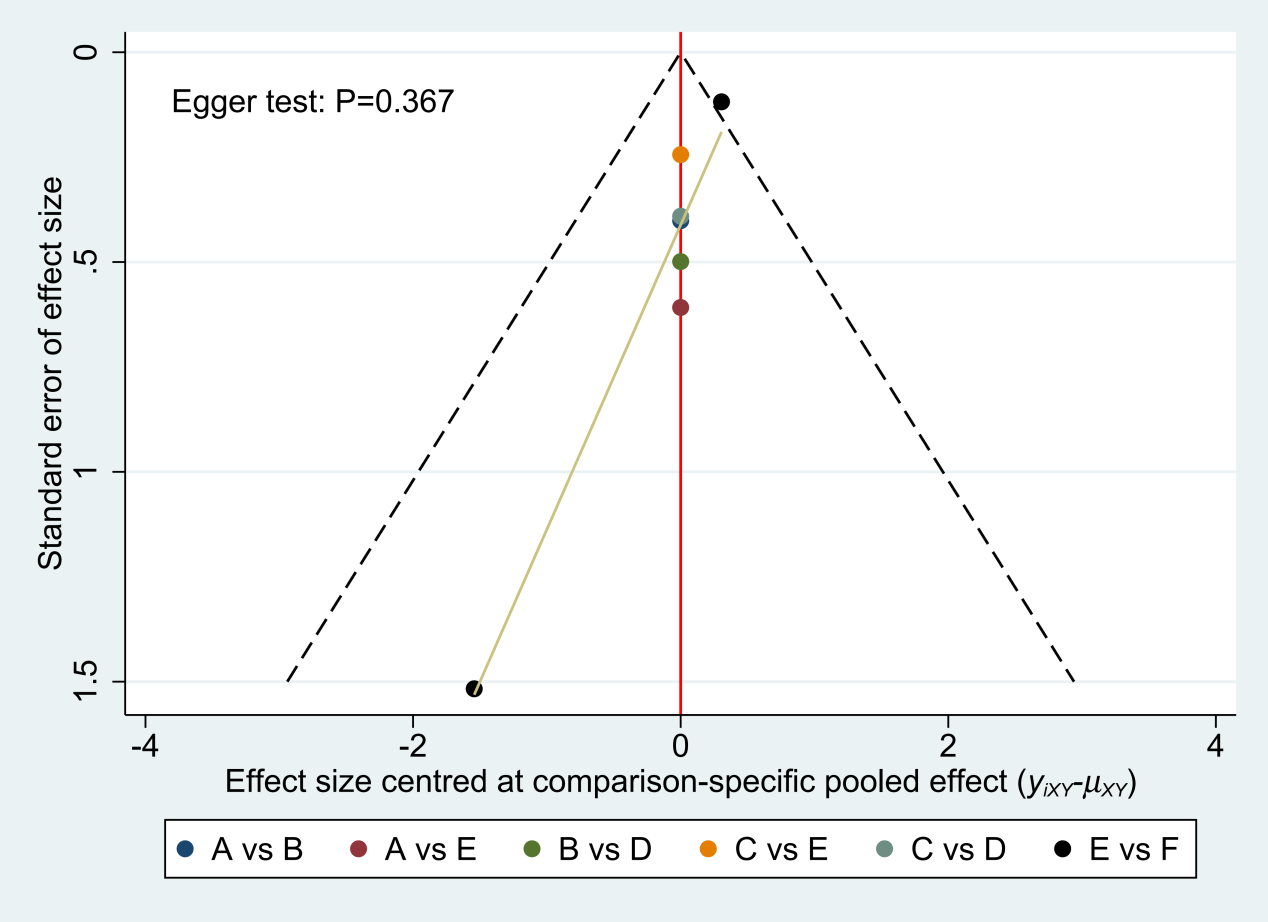


Figure 5.3 The funnel plot of Worsening heart failure. The result of Egger test showed the p=0.367.


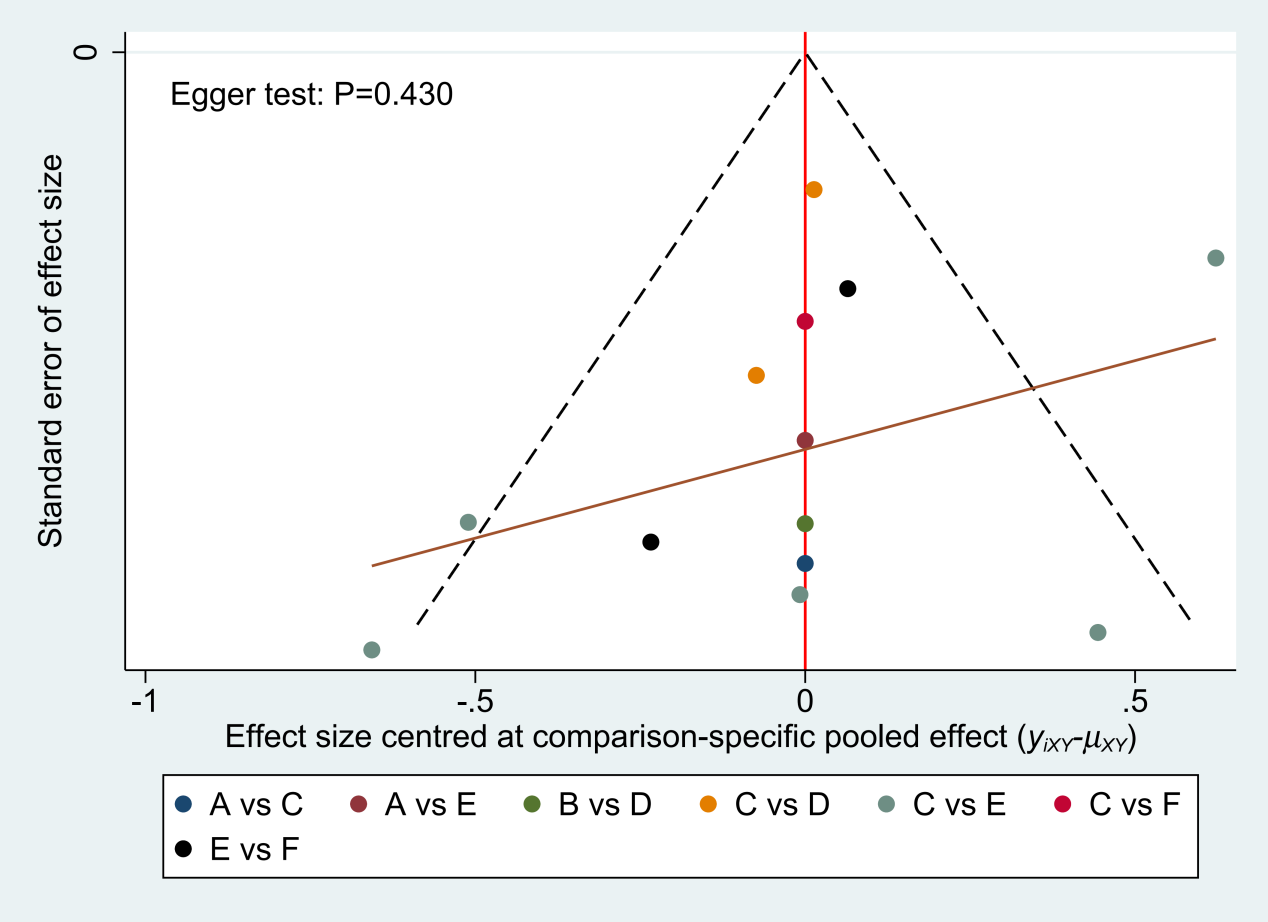


Figure 5.4 The funnel plot of QoL. The result of Egger test showed the p=0.430.


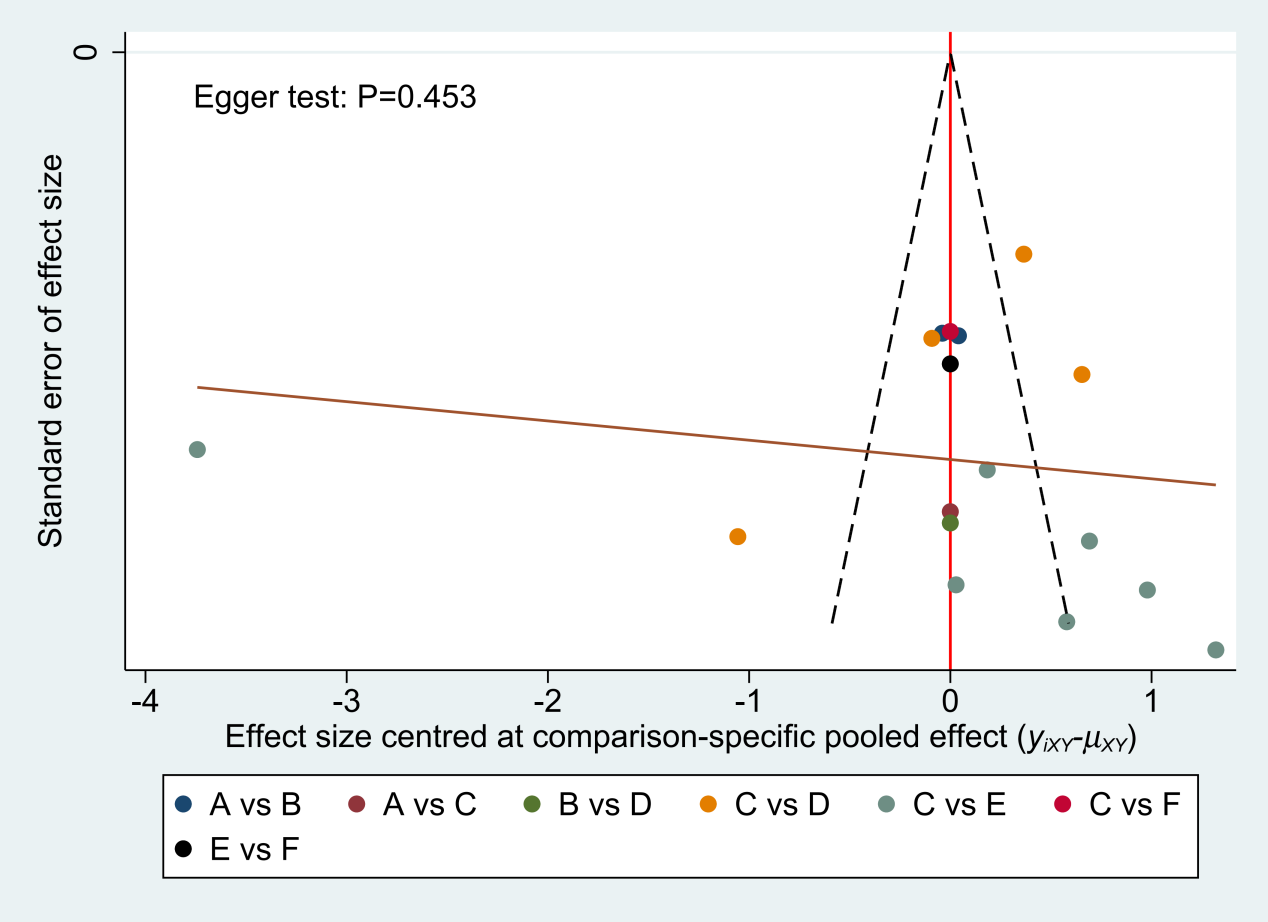


Figure 5.5 The funnel plot of LVEF. The result of Egger test showed the p=0.453.


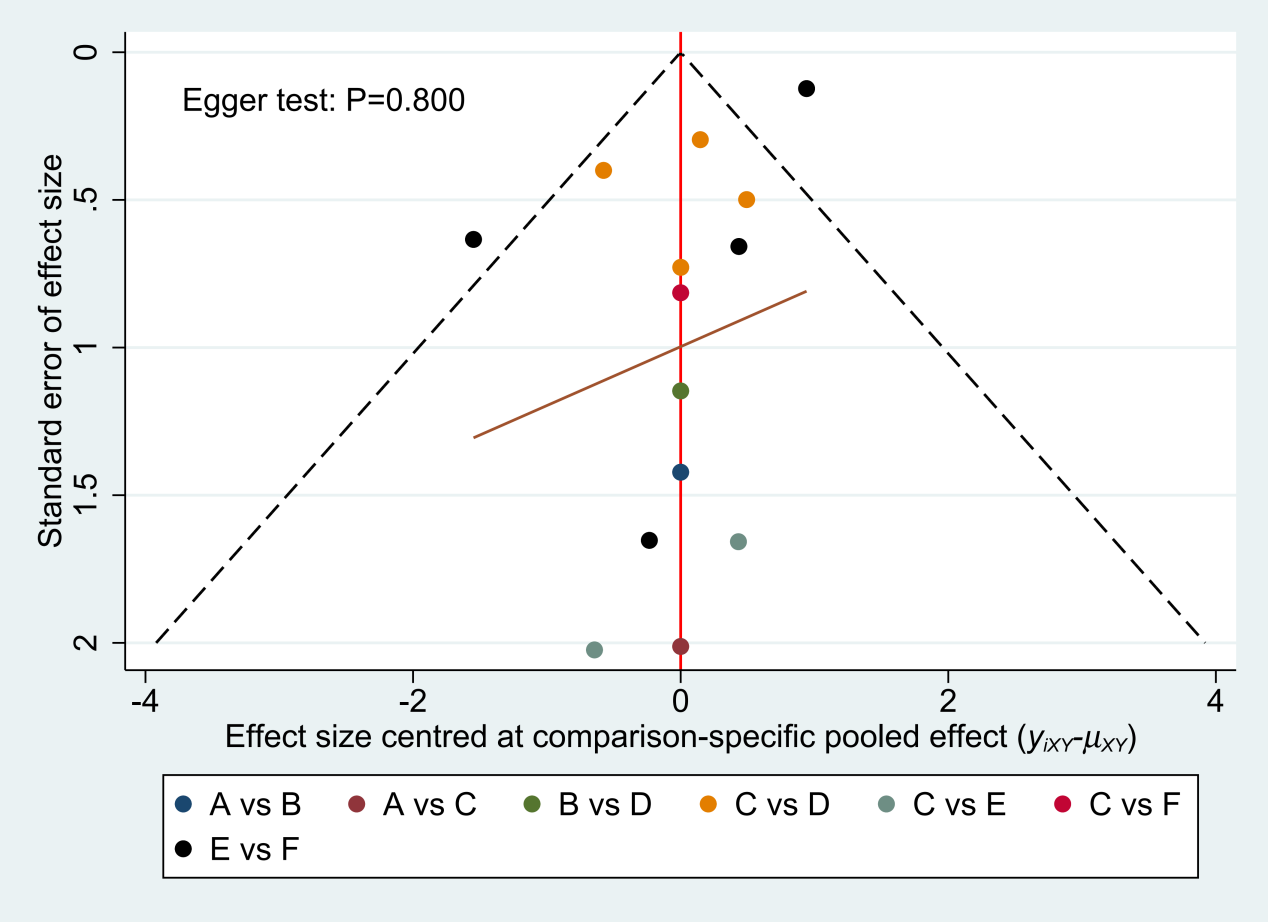


Figure 5.6 The funnel plot of Cardiovascular mortality. The result of Egger test showed the p=0.800.

**Table 5.1. Egger’s regression test for small-study effects**

| **Outcome** | **Regression coefficient** | **Standard error** | **P value** |
| --- | --- | --- | --- |
| All-cause mortality | 0.5186853 | 0.5031169 | 0.316 |
| Heart failure hospitalization | 0.9111647 | 1.029242 | 0.405 |
| Worsening heart failure | 0.9114135 | 0.9198344 | 0.367 |
| QoL | -1.812363 | 2.212187 | 0.430 |
| LVEF | -3.922308 | 5.096026 | 0.453 |
| Cardiovascular mortality | 0.1541886 | 0.5946334 | 0.800 |

# Supplementary 6: Forest plot


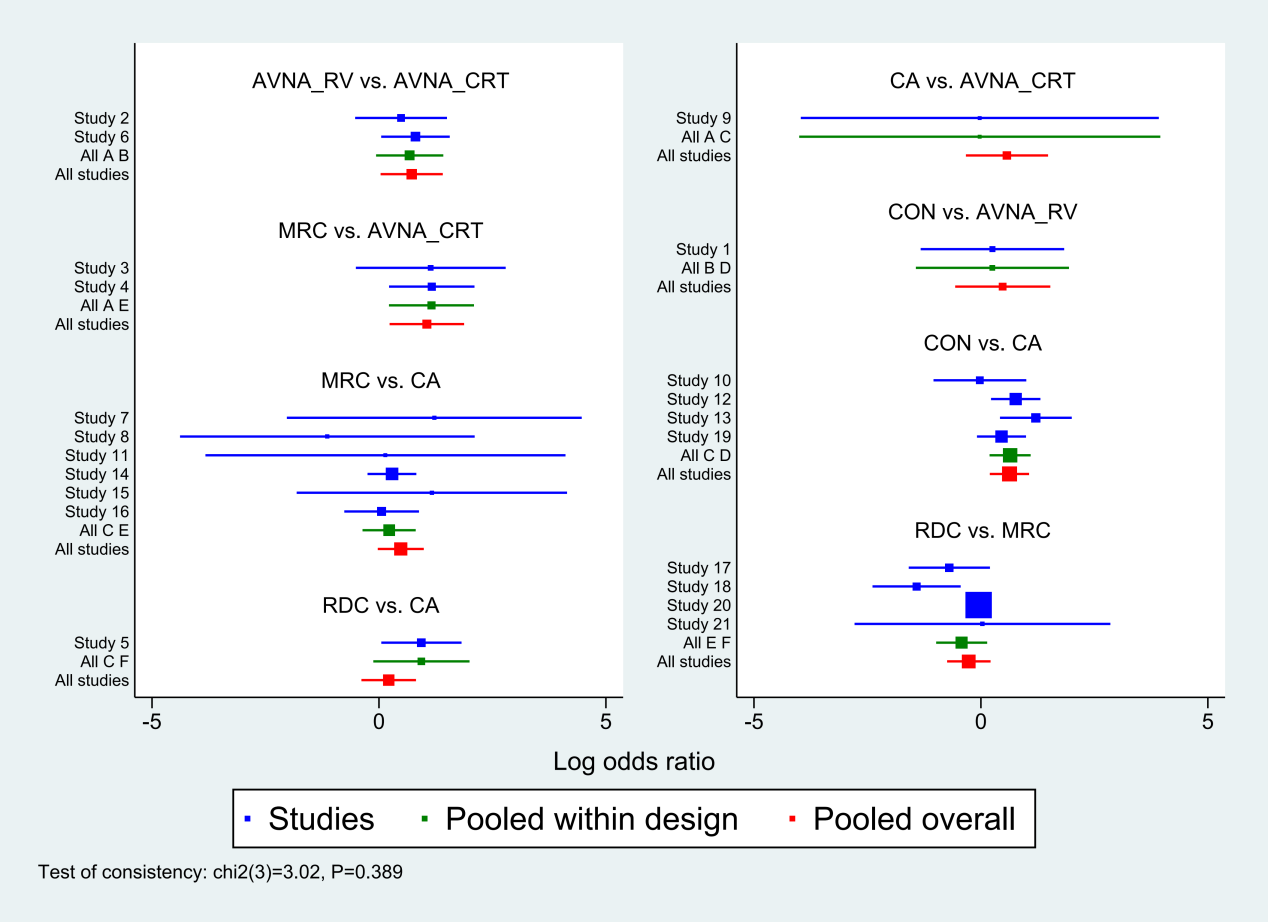


Figure 6.1 The forest plot of All-cause mortality. The result of consistency test showed the p=0.389.


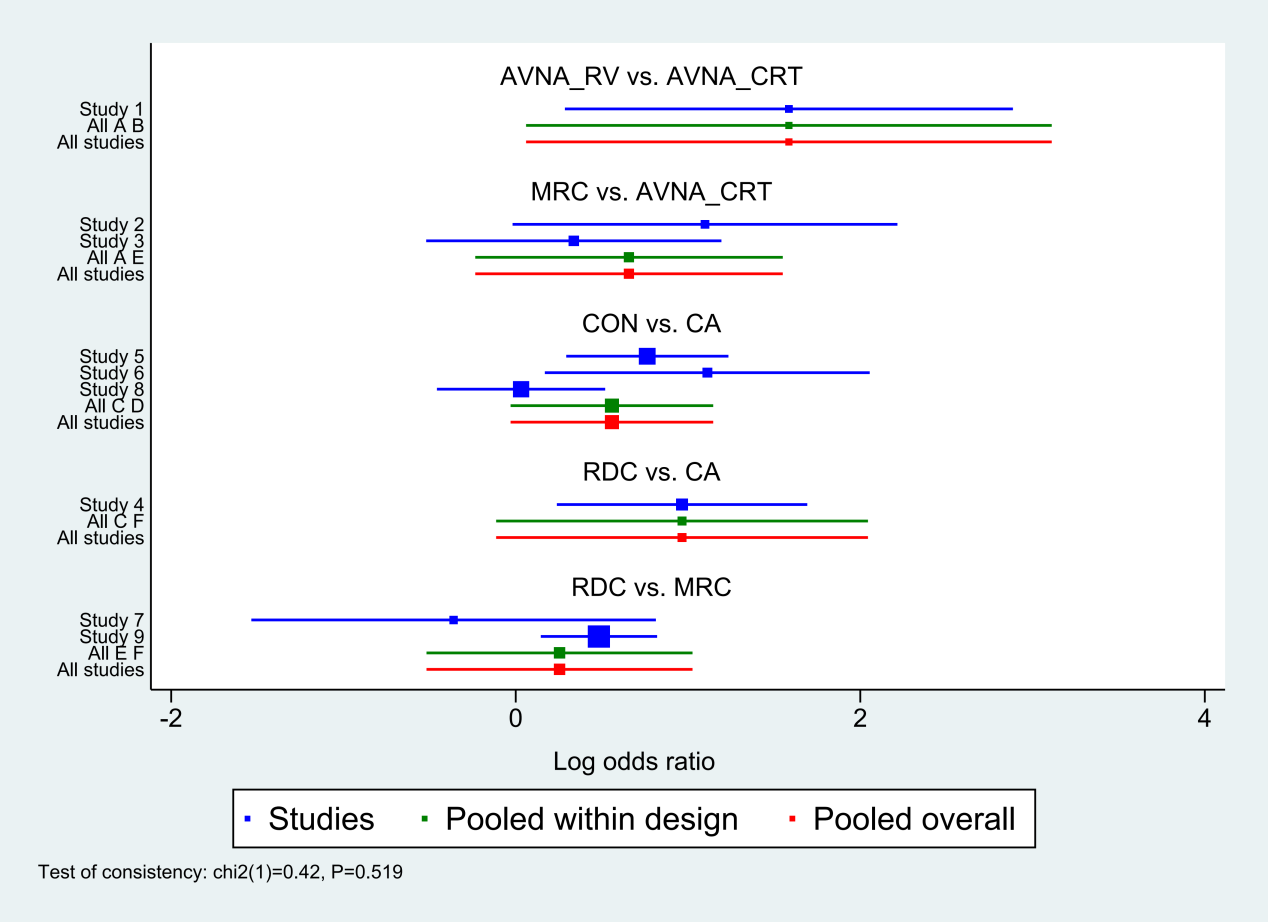


Figure 6.2 The forest plot of Heart failure hospitalization. The result of consistency test showed the p=0.519.


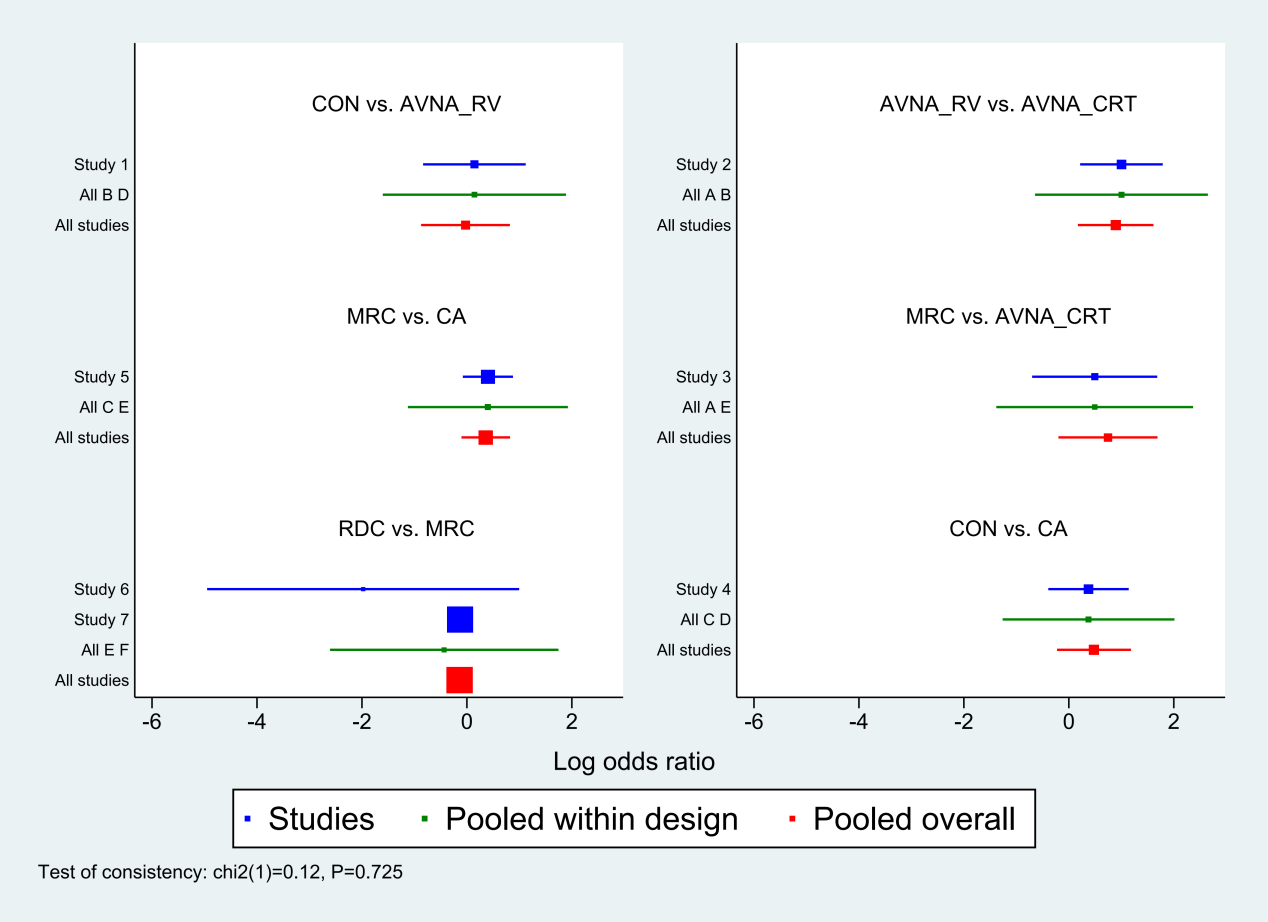


Figure 6.3 The forest plot of Worsening heart failure. The result of consistency test showed the p=0.725.


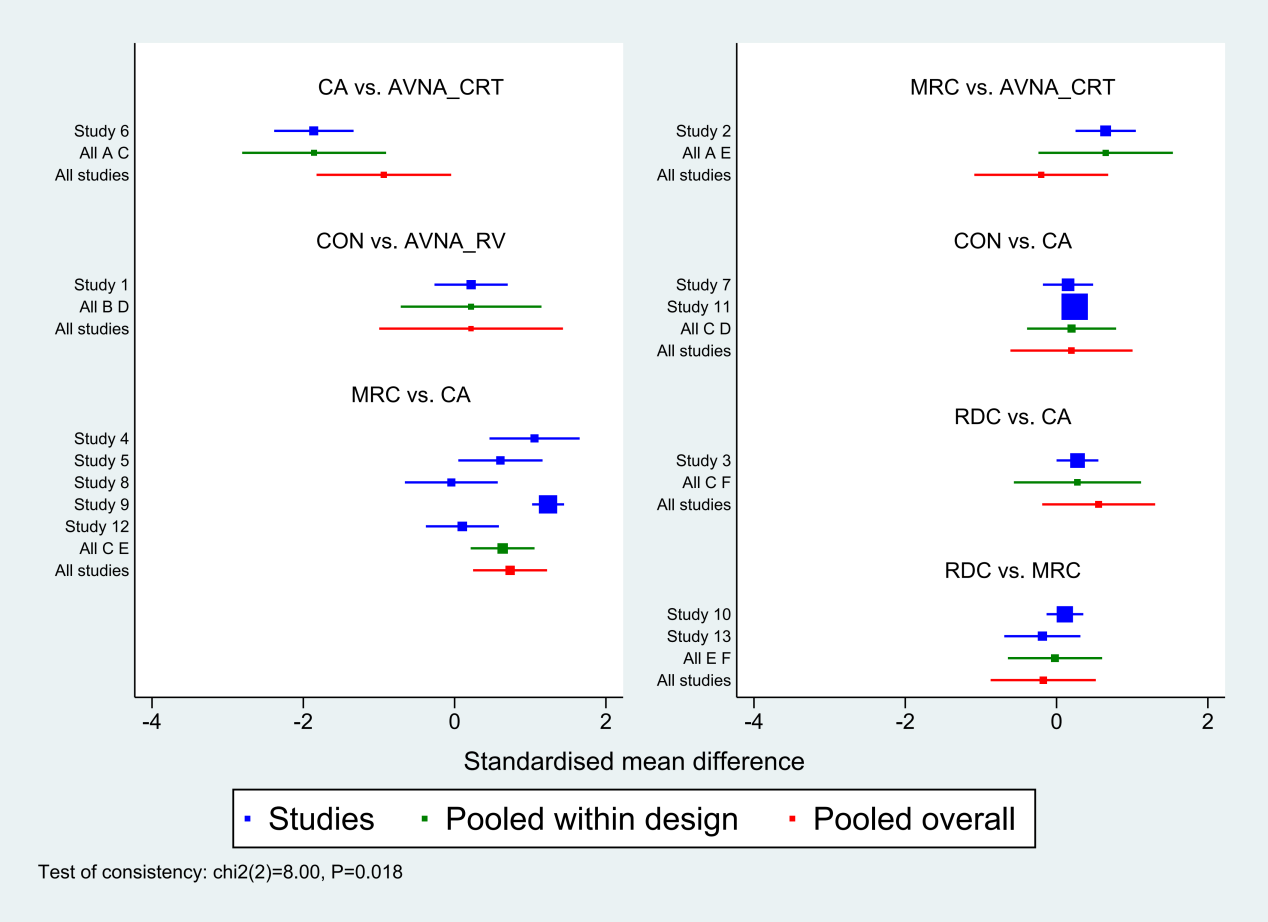


Figure 6.4 The forest plot of QoL. The result of consistency test showed the p=0.018.


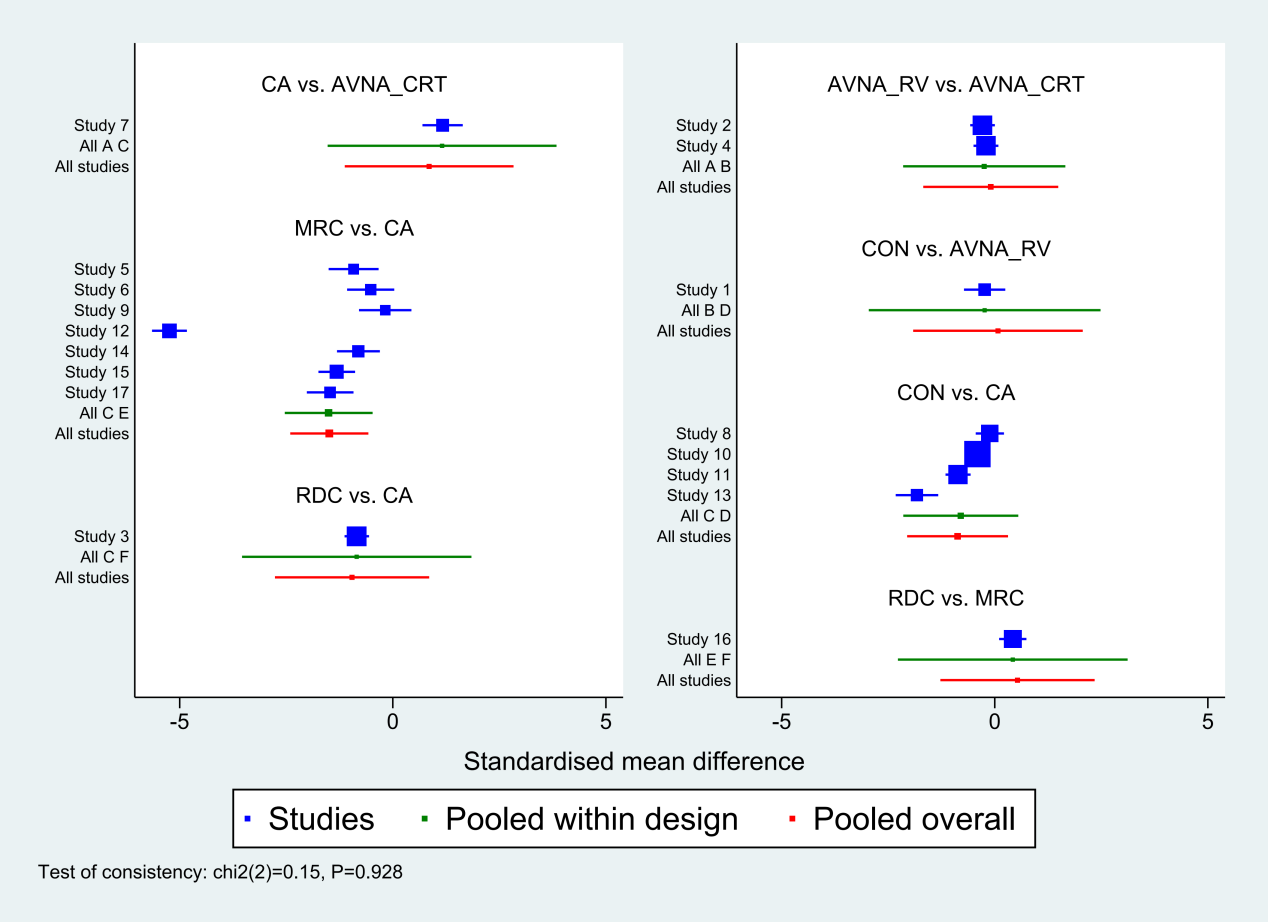


Figure 6.5 The forest plot of LVEF. The result of consistency test showed the p=0.928.


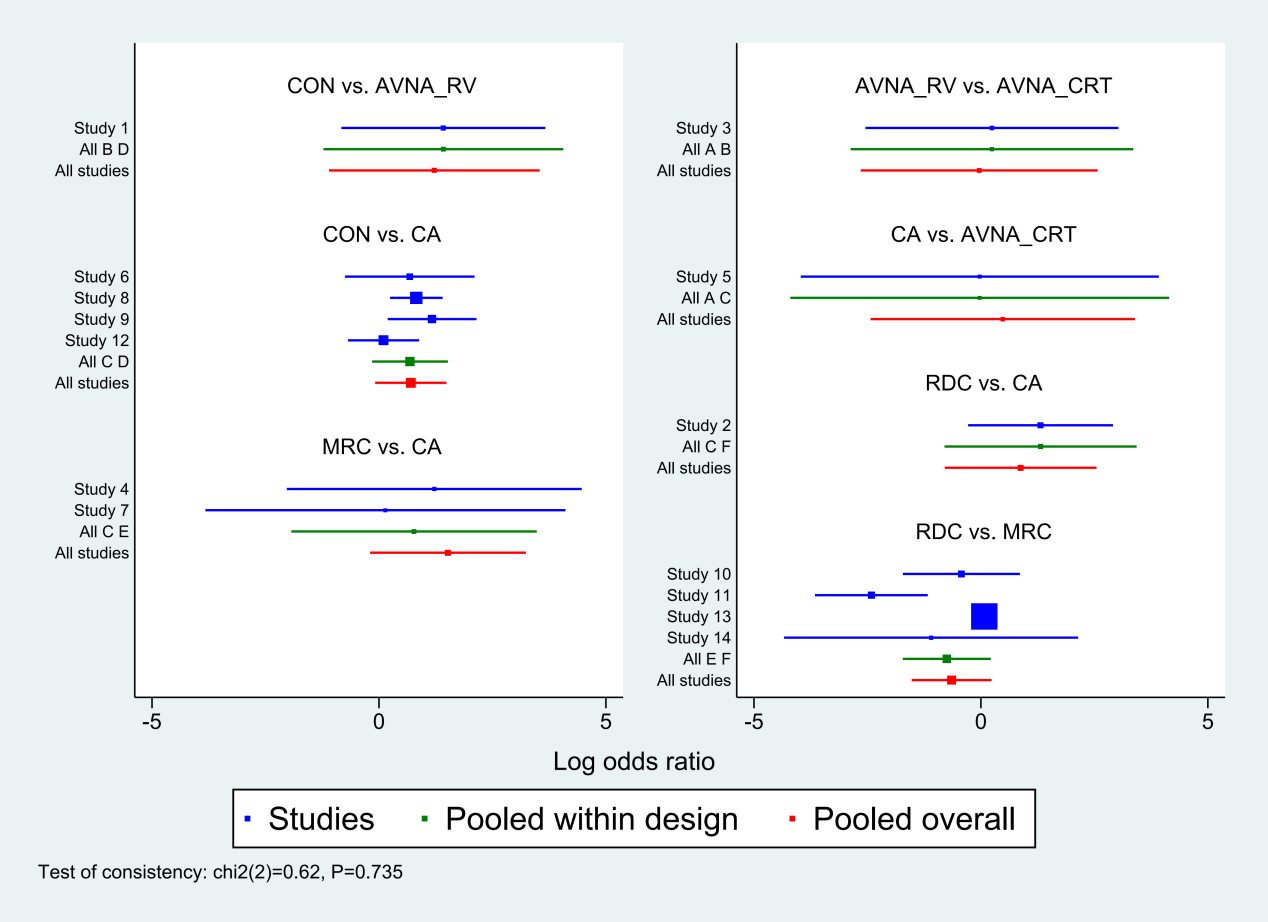


Figure 6.6 The forest plot of Cardiovascular mortality. The result of consistency test showed the p=0.735.

# Supplementary 7: The results of node-splitting

Table 7.1 The results of node-splitting for All-cause mortality.

| **Side** | | **Direct** | | **Indirect** | | **Difference** | | **P>\|z\|** | **tau** |
| --- | --- | --- | --- | --- | --- | --- | --- | --- | --- |
|  |  | **Coef.** | **Std. Err.** | **Coef.** | **Std. Err.** | **Coef.** | **Std. Err.** |  |  |
| A | B | 0.6767635 | 0.3792651 | 1.042566 | 1.040263 | -0.3658025 | 1.106449 | 0.741 | 0.3023484 |
| A | C | -0.0243915 | 2.032382 | 0.6056688 | 0.473964 | -0.6300603 | 2.086916 | 0.763 | 0.2859896 |
| A | E | 1.156863 | 0.4779726 | 0.6821696 | 0.9137286 | 0.4746931 | 1.031226 | 0.645 | 0.2988239 |
| B | D | 0.2537765 | 0.8617215 | 0.6196482 | 0.6940491 | -0.3658716 | 1.106467 | 0.741 | 0.3023487 |
| C | D | 0.6432027 | 0.2320937 | 0.2770265 | 1.081454 | 0.3661762 | 1.10645 | 0.741 | 0.3023462 |
| C | E | 0.2238943 | 0.2873166 | 1.228926 | 0.5062127 | -1.005031 | 0.5824195 | 0.084 | 0.2701716 |
| C | F | 0.9353816 | 0.5330525 | -0.1303297 | 0.3930524 | 1.065711 | 0.6622955 | 0.108 | 0.2844346 |
| E | F | -0.4108516 | 0.2822809 | 0.6546718 | 0.602699 | -1.065523 | 0.66226 | 0.108 | 0.2844263 |

Table 7.2 The results of node-splitting for Heart failure hospitalization.

| **Side** | | **Direct** | | **Indirect** | | **Difference** | | **P>\|z\|** | **tau** |
| --- | --- | --- | --- | --- | --- | --- | --- | --- | --- |
|  |  | **Coef.** | **Std. Err.** | **Coef.** | **Std. Err.** | **Coef.** | **Std. Err.** |  |  |
| A | B | . | . | . | . | . | . | . | . |
| A | E | 0.6577224 | 0.4554768 | 0.262871 | 35.17078 | 0.3948514 | 35.17367 | 0.991 | 0.4070391 |
| C | D | 0.5581362 | 0.3000892 | 0.0633815 | 89.42606 | 0.4947547 | 89.42655 | 0.996 | 0.4070377 |
| C | F | 0.965431 | 0.5506859 | 1.268176 | 42.96385 | -0.3027453 | 42.96731 | 0.994 | 0.4070418 |
| E | F | 0.2541087 | 0.3939886 | -0.1200584 | 37.82231 | 0.3741671 | 37.82418 | 0.992 | 0.4070416 |

Table 7.3 The results of node-splitting for Worsening heart failure.

| **Side** | | **Direct** | | **Indirect** | | **Difference** | | **P>\|z\|** | **tau** |
| --- | --- | --- | --- | --- | --- | --- | --- | --- | --- |
|  |  | **Coef.** | **Std. Err.** | **Coef.** | **Std. Err.** | **Coef.** | **Std. Err.** |  |  |
| A | B | 1.002291 | 0.8395748 | 0.3236882 | 1.732983 | 0.6786033 | 1.925647 | 0.725 | 0.7373731 |
| A | E | 0.4924765 | 0.9551692 | 1.168982 | 1.669802 | -0.6765051 | 1.923691 | 0.725 | 0.7364772 |
| B | D | 0.1430999 | 0.8911511 | -0.5353858 | 1.709785 | 0.6784858 | 1.928087 | 0.725 | 0.7384425 |
| C | D | 0.3749374 | 0.8358067 | 1.052894 | 1.738397 | -0.6779562 | 1.928885 | 0.725 | 0.7387905 |
| C | E | 0.4001518 | 0.777686 | -0.2773336 | 1.764241 | 0.6774854 | 1.928041 | 0.725 | 0.7384126 |
| E | F | -0.1397582 | 0.1178174 | -1.457645 | 133.1526 | 1.317887 | 133.1526 | 0.992 | 2.94E-07 |

Table 7.4 The results of node-splitting for QoL.

| **Side** | | **Direct** | | **Indirect** | | **Difference** | | **P>\|z\|** | **tau** |
| --- | --- | --- | --- | --- | --- | --- | --- | --- | --- |
|  |  | **Coef.** | **Std. Err.** | **Coef.** | **Std. Err.** | **Coef.** | **Std. Err.** |  |  |
| A | C | -1.860474 | 0.4685869 | 0.0579573 | 0.4746943 | -1.918431 | 0.6670145 | 0.004 | 0.3844224 |
| A | E | 0.6492399 | 0.4349131 | -1.265883 | 0.5055479 | 1.915123 | 0.6668793 | 0.004 | 0.3844133 |
| B | D | 0.2180763 | 0.6198107 | -1.470168 | 63.26468 | 1.688244 | 63.26769 | 0.979 | 0.5684324 |
| C | D | 0.1969461 | 0.4121074 | 1.316333 | 25.83121 | -1.119387 | 25.8345 | 0.965 | 0.5684526 |
| C | E | 0.6135893 | 0.2855848 | 1.206689 | 0.5708576 | -0.5931002 | 0.6388239 | 0.353 | 0.5838256 |
| C | F | 0.2762303 | 0.6130164 | 0.7596867 | 0.5237575 | -0.4834565 | 0.8062946 | 0.549 | 0.5965667 |
| E | F | -0.0291493 | 0.4443855 | -0.5121445 | 0.6726156 | 0.4829952 | 0.8062444 | 0.549 | 0.5965596 |

Table 7.5 The results of node-splitting for LVEF.

| **Side** | | **Direct** | | **Indirect** | | **Difference** | | **P>\|z\|** | **tau** |
| --- | --- | --- | --- | --- | --- | --- | --- | --- | --- |
|  |  | **Coef.** | **Std. Err.** | **Coef.** | **Std. Err.** | **Coef.** | **Std. Err.** |  |  |
| A | B | -0.2466636 | 0.9258405 | 0.5968806 | 1.970816 | -0.8435443 | 2.177452 | 0.698 | 1.300919 |
| A | C | 1.168586 | 1.32234 | 0.3244328 | 1.710217 | 0.8441531 | 2.161811 | 0.696 | 1.300136 |
| B | D | -0.2372489 | 1.324307 | 0.6076882 | 1.731155 | -0.8449372 | 2.179607 | 0.698 | 1.301025 |
| C | D | -0.7952065 | 0.6566227 | -1.646027 | 2.081341 | 0.8508206 | 2.182464 | 0.697 | 1.30116 |
| C | E | -1.504719 | 0.5054953 | -1.270304 | 1.858377 | -0.2344152 | 1.925902 | 0.903 | 1.31025 |
| C | F | -0.8450368 | 1.318851 | -1.082026 | 1.413495 | 0.2369892 | 1.933219 | 0.902 | 1.310673 |
| E | F | 0.4225902 | 1.320867 | 0.6596604 | 1.411977 | -0.2370702 | 1.933486 | 0.902 | 1.310687 |

Table 7.6 The results of node-splitting for Cardiovascular mortality.

| **Side** | | **Direct** | | **Indirect** | | **Difference** | | **P>\|z\|** | **tau** |
| --- | --- | --- | --- | --- | --- | --- | --- | --- | --- |
|  |  | **Coef.** | **Std. Err.** | **Coef.** | **Std. Err.** | **Coef.** | **Std. Err.** |  |  |
| A | B | 0.2429462 | 1.574121 | -0.7637689 | 2.534862 | 1.006715 | 2.983853 | 0.736 | 0.6749462 |
| A | C | -0.0243915 | 2.122326 | 0.9716881 | 2.097859 | -0.9960796 | 2.984172 | 0.739 | 0.6748934 |
| B | D | 1.419067 | 1.330842 | 0.4123792 | 2.670961 | 1.006687 | 2.984163 | 0.736 | 0.6749506 |
| C | D | 0.6830622 | 0.4117791 | 1.688864 | 2.956656 | -1.005802 | 2.985193 | 0.736 | 0.6749536 |
| C | E | 0.7739729 | 1.374904 | 2.056164 | 1.172699 | -1.282191 | 1.807816 | 0.478 | 0.689859 |
| C | F | 1.314633 | 1.067457 | 0.0302262 | 1.458753 | 1.284407 | 1.807606 | 0.477 | 0.6899066 |
| E | F | -0.7428414 | 0.4865258 | 0.5409223 | 1.740599 | -1.283764 | 1.808034 | 0.478 | 0.6899005 |

# Supplementary 8: Sensitivity analysis excluding studies with some concerns

| Outcome | Comparison | Main analysis | Estimated sensitivity analysis  (low-risk only) | Interpretation |
| --- | --- | --- | --- | --- |
| All-cause mortality | CA vs CON | OR 0.53 (0.35 to 0.82) | OR 0.58 (0.37 to 0.91) | Direction unchanged; effect modestly attenuated but remains statistically significant |
| All-cause mortality | AVNA_CRT vs MRC | OR 0.35 (0.15 to 0.79) | OR 0.40 (0.19 to 0.87) | Direction unchanged; benefit remains statistically significant |
| All-cause mortality | AVNA_CRT vs CON | OR 0.30 (0.12 to 0.78) | OR 0.43 (0.18 to 1.03) | Direction unchanged, but precision reduced and statistical significance lost |
| All-cause mortality | AVNA_CRT vs AVNA_RV | OR 0.49 (0.25 to 0.96) | NA | AVNA_RV node removed after exclusion of studies with some concerns |
| HF hospitalization | AVNA_CRT vs AVNA_RV | OR 0.20 (0.04 to 0.94) | NA | AVNA_RV node removed after exclusion of studies with some concerns |
| HF hospitalization | CA vs CON | Not highlighted in main text | OR 0.62 (0.33 to 1.16) | Direction favors CA, but not statistically significant |
| HF hospitalization | AVNA_CRT vs MRC | Not highlighted in main text | OR 0.56 (0.28 to 1.13) | Direction favors AVNA_CRT, but not statistically significant |
| HF hospitalization | AVNA_CRT vs CON | Not highlighted in main text | OR 0.46 (0.19 to 1.12) | Direction favors AVNA_CRT, but not statistically significant |
